# Targeted Repair of Spinal Cord Injury Based on miRNA‐124‐3p–Loaded Mesoporous Silica Camouflaged by Stem Cell Membrane Modified with Rabies Virus Glycoprotein

**DOI:** 10.1002/advs.202309305

**Published:** 2024-03-21

**Authors:** Xiangchuang Fan, Lusen Shi, Zimeng Yang, Yiwei Li, Chi Zhang, Baoshuai Bai, Lu Chen, Elzat Elham‐Yilizati Yilihamu, Zhangyang Qi, Wenxiang Li, Peng Xiao, Mingshan Liu, Jichuan Qiu, Fan Yang, Ning Ran, Yifan Shang, Jiaxing Liu, Tehan Zhang, Xiaohong Kong, Hong Liu, Hengxing Zhou, Shiqing Feng

**Affiliations:** ^1^ Department of Orthopaedics Qilu Hospital of Shandong University Shandong University Centre for Orthopaedics Cheeloo College of Medicine Shandong University Jinan 250012 P. R. China; ^2^ State Key Laboratory of Crystal Materials Shandong University Jinan 250100 P. R. China; ^3^ Key Laboratory Experimental Teratology of the Ministry of Education Department of Biochemistry and Molecular Biology School of Basic Medical Sciences Cheeloo College of Medicine Shandong University Jinan 250012 P. R. China; ^4^ Advanced Medical Research Institute Shandong University Jinan 250012 P. R. China; ^5^ The Second Hospital of Shandong University Cheeloo College of Medicine Shandong University Jinan 250033 P. R. China; ^6^ Hefei National Laboratory Jinan Branch Jinan Institute of Quantum Technology Jinan 250101 P. R. China; ^7^ Department of Orthopaedics Tianjin Medical University General Hospital International Science and Technology Cooperation Base of Spinal Cord Injury Tianjin Key Laboratory of Spine and Spinal Cord Tianjin Medical University Tianjin 300052 P.R. China

**Keywords:** axonal regeneration, microRNA‐124‐3p, spinal cord injury, targeted repair

## Abstract

Spinal cord injury (SCI) has no effective treatment modalities. It faces a significant global therapeutical challenge, given its features of poor axon regeneration, progressive local inflammation, and inefficient systemic drug delivery due to the blood–spinal cord barrier (BSCB). To address these challenges, a new nano complex that achieves targeted drug delivery to the damaged spinal cord is proposed, which contains a mesoporous silica nanoparticle core loaded with microRNA and a cloaking layer of human umbilical cord mesenchymal stem cell membrane modified with rabies virus glycoprotein (RVG). The nano complex more readily crosses the damaged BSCB with its exosome‐resembling properties, including appropriate size and a low‐immunogenic cell membrane disguise and accumulates in the injury center because of RVG, where it releases abundant microRNAs to elicit axon sprouting and rehabilitate the inflammatory microenvironment. Culturing with nano complexes promotes axonal growth in neurons and M2 polarization in microglia. Furthermore, it showed that SCI mice treated with this nano complex by tail vein injection display significant improvement in axon regrowth, microenvironment regulation, and functional restoration. The efficacy and biocompatibility of the targeted delivery of microRNA by nano complexes demonstrate their immense potential as a noninvasive treatment for SCI.

## Graphical abstract

The present research reports a novel strategy to repair spinal cord injury based on microRNA‐124‐3p loaded mesoporous silica nanoparticle camouflaged with human umbilical cord stem cell membrane. This nano complex shows tremendous potential in targeted delivery while addressing challenges posed by the blood‐spinal cord barrier and immunologic clearance. Multifaceted therapeutic effects are demonstrated in and ex vivo.

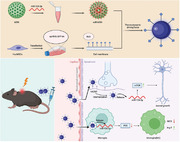

## 1 Introduction

Spinal cord injury (SCI) is a disastrous event for patients that often means lifetime comorbidities, like dyskinesia, sensory dysfunction, and even death.^[^ ^1^, ^2^, ^3^ ^]^ Care of patients with SCI takes a heavy economic toll on families and society. However, seeking a cure involves strategic frustrations and ineffective treatment interventions.^[^ ^1^, ^4^, ^5^ ^]^ In the context of the incredible global incidence of 10.4–83.0 cases/million/year, growing research endeavors are undertaken to explore novel therapeutic approaches, especially from the pathophysiological perspective.^[^ ^6^, ^7^ ^]^

The inflammatory microenvironment forming after SCI‐related axon disruption and myelin necrosis involves the rapid activation and aggregation of microglia/macrophages that play a pivotal role in modulating inflammatory microenvironment and affecting secondary injury.^[^ ^8^ ^]^ Therefore, promoting axon regeneration and modulating the inflammatory microenvironment are instrumental in SCI treatment. Research strategies for elongating axons and regulating the inflammatory microenvironment in SCI include scaffold biomaterials, medication, and targeted exosomes.^[^ ^8^, ^9^, ^10^, ^11^ ^]^ These treatment methods have limitations. The invasive implantation of various scaffold biomaterials, like hydrogels, carries the risk of secondary injury.^[^ ^9^, ^12^ ^]^ Drug application is complicated by the need to achieve sufficient delivery to the damaged spinal cord because most drug channels are limited by the blood–spinal cord barrier (BSCB).^[^ ^13^ ^]^ Exosomes derived from divergent cells contribute to intercellular communication by allowing the exchange of proteins and genetic materials between cells. They are also permitted across the BSCB to reach the injured spinal cord while escaping phagocytosis under low immunogenicity.^[^ ^13^, ^14^, ^15^ ^]^ Nevertheless, the application of exosomes is hampered by limited production from cell culture and complicated purification processes. In contrast, as a critical content of exosomes, microRNAs (miRNAs) are more accessible to synthesize artificially and are more cost‐friendly. As small non‐coding RNA molecules ≈20–22 nucleotides long,^[^ ^16^ ^]^ miRNAs regulate various transcriptions and have exhibited considerable therapeutic functions.^[^ ^17^, ^18^ ^]^ Previous studies have demonstrated that miRNA‐124‐3p facilitates neural recovery after SCI by stimulating axon growth and microglia polarization to the anti‐inflammatory phenotype, which indicates its potential application in overcoming the obstacles in SCI treatment.^[^ ^16^, ^19^, ^20^, ^21^ ^]^ An efficient delivery system for transporting miRNA‐124‐3p to the center of the SCI needs to be established to achieve suitable therapeutic outcomes.

In recent years, increased efforts have been committed to constructing nano complex–based drug delivery systems with improved efficacy.^[^ ^13^ ^]^ For instance, mesoporous silica nanoparticles (MSNs), which possess high miRNA load capacity and satisfactory biodegradability, have emerged as effective vehicles for the in vivo transportation of miRNA.^[^ ^22^ ^]^ However, emerging difficulties, such as the degradation of miRNA by body fluids and the excessive clearance of MSNs by peripheral immune cells, afflict this new transport system. One solution is to cloak MSNs with a hypoimmunogenic stem cell membrane shielding the contents from immunological elimination and insulating the miRNA content from degradation.^[^ ^23^ ^]^ Lacking targeting ability, the cell membrane‐camouflaged MSNs present another challenge. Although the damaged BSCB allows the extravascular access of appropriately sized particles, such as exosomes or similarly sized MSNs, the scarcity of localized specific binding sites for these particles often results in low drug retention and accumulation rate.^[^ ^24^ ^]^ Thus, to achieve favorable repair of SCI with miRNA‐124‐3p, three requirements need to be satisfied simultaneously: i) efficient miRNA loading, ii) hypoimmunogenic exosome‐like particles to cross the BSCB, and iii) potent spinal cord/neuron‐targeting ability.

One promising strategy for overcoming these challenges involves modifying stem cell membrane using proteins with a specific binding effect for the targeted delivery of drugs. Rabies virus glycoprotein (RVG) serves as an ideal candidate owning excellent neuron‐targeting ability with specific binding to the nicotinic acetylcholine receptors (nAChR) on neurons.^[^ ^25^, ^26^ ^]^ In this study, we transfected human umbilical cord mesenchymal stem cells (HucMSCs) with lentivirus (sp‐RVG‐GFP‐tm) to induce the overexpression of the neuron‐targeting RVG on the membrane. We then assembled the resultant human umbilical cord mesenchymal stem cell membrane (HucMSCM) onto the surface of the MSN loaded with miRNA‐124‐3p. The resultant nano complexes demonstrated low immunogenicity and an exosome‐like particle size to infiltrate the damaged BSCB and achieve the efficient, targeted delivery of miRNA‐124‐3p to the spinal cord in an SCI mouse model. After administration of these nano complexes by tail vein injection, we found that the nano complexes effectively promoted axon regrowth, modulated the local inflammatory microenvironment, and enhanced the functional recovery of SCI without causing treatment‐related secondary injury. The present study thus describes this novel noninvasive biological repair strategy and presents new possibilities for the clinical translation of nanomaterial‐delivered miRNAs in SCI therapy.

## 2 Results and Discussion

### 2.1 Construction of HucMSCM‐Camouflaged Nano Complex

**Scheme 1:**
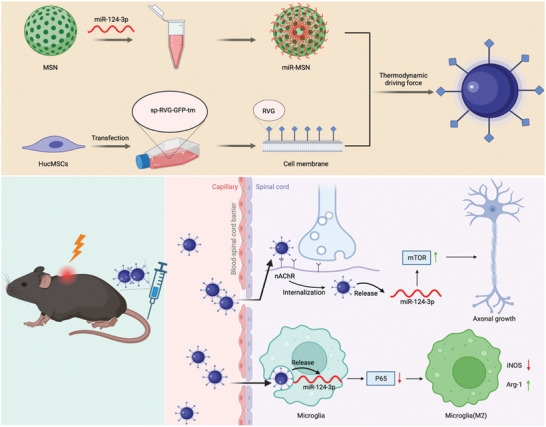
Scheme of fabricating the human umbilical cord mesenchymal stem cell membrane (HucMSCM)‐camouflaged nano complex for miRNA‐mediated spinal cord injury (SCI) repair. The synthetic steps for miRNA‐124‐3p–loaded mesoporous silica nanoparticle (miR‐MSN), rabies virus glycoprotein‐modified HucMSCM, and self‐assembly of CM‐miR‐MSN nano complex are shown. Administrated by tail vein injection in an SCI mouse model, the nano complex can cross the blood‐spinal cord barrier and target the injured spinal cord. Upon entering neurons and microglia, the CM‐miR‐MSN releases miRNA‐124‐3p into the cytoplasm to boost axon regrowth and modulate the local inflammatory microenvironment through the mTOR and P65 pathways, respectively. Original figure created using BioRender.com.

Designing nano complexes with the practical employment of RVG facilitates the realization of targeted SCI repair (**Scheme** **1**). The fabrication of our nano complex included three steps: i) synthesis of the miRNA‐124‐3p–loaded MSN (miR‐MSN); ii) preparation of the HucMSCM modified with RVG and isolation of the resulting cell membrane (CM); and iii) self‐assembly of the CM onto the miR‐MSN surface to form the CM‐camouflaged miR‐MSN (CM‐miR‐MSN) nano complexes. The first step leveraged the electrostatic interactions between the negatively charged miRNA and positively charged, amino‐carrying MSN. Given its excellent nucleic acid loading and delivery capacity, we packed MSN with miRNA.^[^ ^22^, ^27^, ^28^, ^29^ ^]^ The preparation of the CM was accomplished by transfecting HucMSCs with sp‐RVG‐GFP‐tm. Upon entry into susceptible cells, these pseudoviruses can only replicate once, unlike wild‐type (WT) viruses, which often replicate multiple times. Additionally, pseudoviruses also lack the virulent components of their parent virus, which practically eliminates the possibility that these virus particles could cause an active infection in an exposed individual.^[^ ^25^, ^26^ ^]^ The transfection safely enabled RVG expression on HucMSCM. Lastly, the CM was isolated and self‐assembled onto the miR‐MSN surface by thermodynamic force, in conjunction with the interactions at the interface, mainly between membrane phospholipids and proteins and the amino groups on the miR‐MSN.^[^ ^23^, ^30^, ^31^ ^]^ The resulting CM‐miR‐MSN complexes were administered by tail vein injection to induce SCI repair (Scheme 1). The complexes bound onto the nAChR on neurons via the neurospecificity of RVG expressed on the CM or by aggregated microglia phagocytosis. Thereafter, the nano complexes began releasing miRNAs into the cytoplasm. In neurons, it boosted axonal sprouting through the mTOR pathway, whereas in microglia, it promoted polarization toward the M2 phenotype via the NF‐κB pathway (Scheme 1).

### 2.2 Fabrication and Characterization of the Nano Complexes

**Figure 1:**
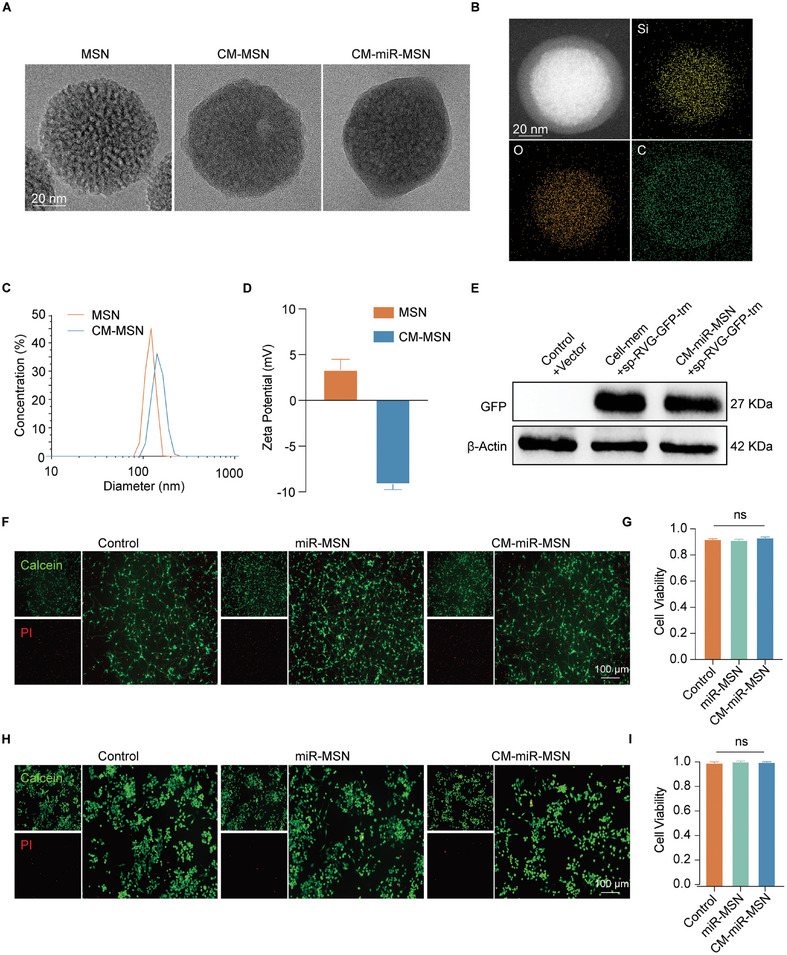
Construction and characterization of the nano complex. A) Transmission electron microscopy images of the mesoporous silica nanoparticle (MSN), cell membrane (CM)‐MSN, and CM‐miR‐MSN. Scale bar: 20 nm. B) Elemental mapping of CM‐MSN. Scale bar: 20 nm. C) Size distribution and D) surface charge of MSN and CM‐MSN measured by dynamic light scattering and zeta potential, respectively. E) Western blot of vector‐transfected cell membrane (control + vector), rabies virus glycoprotein‐overexpressing cell membrane (cell‐mem+sp‐RVG‐GFP‐tm), and CM‐miR‐MSN (CM‐miR‐MSN+sp‐RVG‐GFP‐tm). F) Live/dead staining of neurons (Scale bar: 100 µm) and G) the corresponding analysis performed using one‐way analysis of variance (ANOVA). H) Live/dead staining of BV2 (Scale bar: 100 µm) and I) the corresponding analysis performed using one‐way ANOVA; ns, not significant (p > 0.05).

A growing body of research on nanoparticles has advanced their applications in SCI repair.^[^ ^32^, ^33^ ^]^ Among conventional nanocarriers, MSNs emerge as advantageous vehicles for drug delivery because of their tailorable mesoporous structure and high specific surface area.^[^ ^34^ ^]^ With their extensive nanopores, MSNs offer a more favorable option for controlled and localized drug delivery.^[^ ^35^ ^]^ We modified the MSN with amino groups to generate a positive charge on the MSN surface to ensure effective miRNA loading and CM camouflage. The positive charge facilitates both the loading of negatively charged miRNAs into its mesopores and the cloaking by the negatively charged CM.^[^ ^31^ ^]^ Transmission electron microscopy (TEM) images indicate that the resultant MSNs were spherical mesoporous nanoparticles with diameters ranging from 80 to 120 nm. (**Figure** **1**A). Next, the membrane of transfected HucMSCs was isolated. By placing the materials for assembly in an ultrasonic water bath,^[^ ^36^ ^]^ the CM enveloped the MSN to form a CM‐camouflaged MSN (CM‐MSN). TEM imaging shows that the obtained CM‐MSNs were spherical particles with a complete cloaking layer. The CM‐MSN and miRNA‐loaded CM‐miR‐MSN were highly morphologically similar, suggesting that the miRNA complexation hardly caused any morphological changes to the nano complex. In addition, elemental mapping images visualized distributions of Si, O, and C in CM‐MSN nanoparticles (Figure 1B). Si and O elements were primarily present in the MSN core, whereas the membrane layer held most of the C element. The CM‐miR‐MSN is 90–150 nm in diameter (Figure 1C) within the natural exosome size range, guaranteeing sufficient passage across the BSCB.^[^ ^9^ ^]^ Zeta potential analysis revealed that after CM cloaking, the particle surface changed from positive to negative, suggesting successful CM assembly (Figure 1D). Moreover, to verify the biodegradability of the MSN, we incubated MSNs in fetal bovine serum (FBS) for 12 and 24 h. The MSNs completely degraded in 24 h in FBS (Figure S1, Supporting Information), suggesting the efficacy in miRNA release and low immune‐related side effects in vivo. Also, gradual miRNA release by both miR‐MSN and CM‐miR‐MSN is directly confirmed, as indicated by the miRNA release curve detected in an acidic environment of a lysosomal pH. The release of miRNA at pH 7.0 was also tested. The curve suggests fewer releases of miRNAs, indicating the nano complex was less easy to release in normal conditions but mainly in lysosomes (Figure S2, Supporting Information). RVG has proven satisfactory neuron‐targeting properties.^[^ ^9^ ^]^ Therefore, we assembled RVG on CM by transfecting HucMSCs with lentivirus (sp‐RVG‐GFP‐tm). Western blot analysis of proteins extracted from the resulting HucMSCM and CM‐miR‐MSN confirmed the existence of GFP‐RVG in both the HucMSCM and CM‐miR‐MSN (Figure 1E), thus ensuring RVG expression on the HucMSCM.

Next, we evaluated the biocompatibility of the nano complex on primary neurons and BV2 cells (a microglia cell line). First, neurons harvested from the brain cortex of C57BL/6 mouse embryos were co‐incubated with phosphate‐buffered saline (PBS), miR‐MSN, or CM‐miR‐MSN for 72 h. The live/dead staining showed no difference between the three groups (Figure 1F,G), indicating that the nano complexes had high biocompatibility with primary neurons. Likewise, live/dead staining with BV2 cells revealed no differences among the groups, confirming biocompatibility (Figure 1H,I). We then performed Cell Counting Kit‐8 (CCK‐8) assays with primary neurons and BV2 cells, further confirming the nano complexes’ biocompatibility (Figure S3, Supporting Information). These results collectively validated that the nano complex possesses the prerequisite biocompatibility for further experiments.

### 2.3 Uptake of Nano Complexes by Neurons and Promotion of Axon Growth

**Figure 2:**
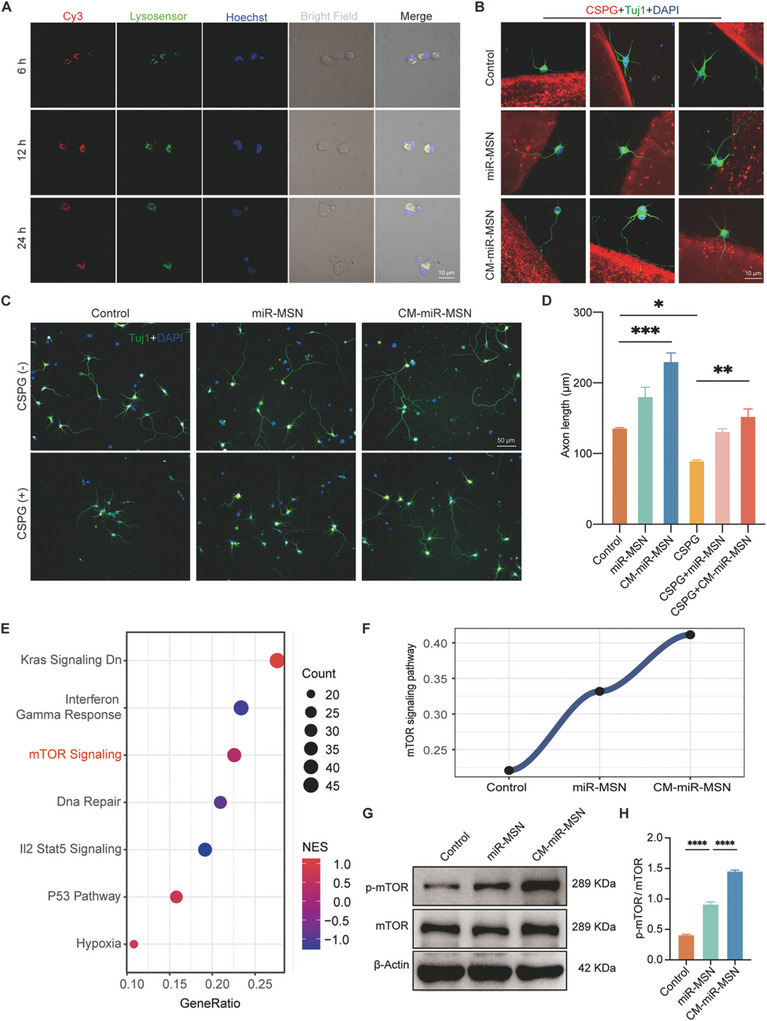
Uptake of nano complexes by neurons and axon elongation evaluated in vitro. A) Representative fluorescence images of CM‐miR‐MSN, lysosomes, and nuclei; bright‐field images and merged images of neurons cultured with CM‐miR‐MSN for 6, 12, and 24 h. The miRNA was stained with Cy3 (red), lysosomes with Lysosensor (green), and nuclei with Hoechst (blue). Scale bar: 10 µm. B) Representative images of primary cortical neurons crossing the chondroitin sulfate proteoglycan (CSPG)‐spot barrier after miR‐MSN and CM‐miR‐MSN phagocytosis. Scale bar: 10 µm. C) Representative images of axon length after CM‐MSN and CM‐miR‐MSN treatment. Tuj1 (green) labels neurons and DAPI (blue) labels nuclei. Scale bar: 50 µm. D) Quantification of axon length (Control: n = 98; miR‐MSN: n = 64; CM‐miR‐MSN: n = 70; CSPG: n = 27, CSPG + miR‐MSN: n = 25; CSPG + CM‐miR‐MSN: n = 26; *p < 0.05, **p < 0.01, and ***p < 0.001, one‐way analysis of variance [ANOVA]). E) Gene ratio analysis and F) mTOR signaling pathway expression expected by RNA‐seq. G) The expression of p‐mTOR and mTOR determined by western blot in each group. H) Quantitative analysis of the p‐mTOR/mTOR ratio. Statistical analysis was performed using one‐way analysis of variance; ****p < 0.0001.

We investigated the specific cellular uptake of neurons in response to the nano complexes by separately incubating Cy3‐marked‐miRNA–loaded miR‐MSN and CM‐miR‐MSN with neurons for 6, 12, and 24 h, and then capturing images using a laser scanning confocal microscope. After 6 h the coincidence of Cy3 fluorescent signals with the lysosomes indicated by Lysosensor occurred in both groups (**Figure** **2**A; Figure S4, Supporting Information). However, the Cy3 signals were much more prominent in the CM‐miR‐MSN group, possibly due to the cloaking of CM that enhanced neuronal uptake.

The significant barriers restricting axonal regeneration after SCI include inflammatory response and glial scars,^[^ ^8^ ^]^ whose main components are reactive astrocytes and chondroitin sulfate proteoglycans (CSPGs), an extracellular matrix component.^[^ ^37^, ^38^ ^]^ Therefore, we explored whether the nano complex can counteract the CSPG‐mediated inhibitory effect on axon elongation and activate the innate growing ability of neurons. We used axonal guidance spot assays to determine whether this nano complex could encourage axons to grow toward the CSPG‐spot barrier. Neurons were incubated with PBS, miR‐MSN, or CM‐miR‐MSN on a CSPG‐spotted coverslip. miR‐MSN and CM‐miR‐MSN enabled cortex neurons to elongate through the CSPG barrier. In contrast, the control group did not (Figure 2B), confirming that the nano complexes could satisfactorily enhance axon growth. Previous studies have reported the inhibitive impact that neuronal medium with CSPG exerts on axon outgrowth in primary neurons.^[^ ^39^ ^]^ We incubated primary cortex neurons on CSPG+ and CSPG− media and added PBS, miR‐MSN, or CM‐miR‐MSN. The average axon length in each medium at 3 days after incubation indicated that CSPG significantly reduced average axon elongation length, while the application of the nano complexes significantly increased axon lengths in both CSPG− and CSPG+ media, confirming that CM‐miR‐MSN can promote axon sprouting of cortex neurons in a CSPG‐mediated inhibitory environment (Figure 2C,D). Altogether, these data demonstrated that the miRNA‐124‐3p–loaded nano complexes alleviate CSPG‐mediated neuron inhibition and elicits axon regeneration in vitro.

To investigate the mechanism of promoting neuronal axon elongation by miRNA‐124‐3p, we sequenced RNA from the control, miR‐MSN, and CM‐miR‐MSN groups. Subsequently, we performed gene set enrichment analysis (GSEA) on the control and CM‐miR‐MSN groups using RNA‐seq data and observed the activation of the mTOR signaling pathway (Figure 2E,F). According to previous studies, the mTOR pathway plays a crucial role in the nervous system, and activation of the mTOR pathway can significantly promote axon growth.^[^ ^40^, ^41^, ^42^ ^]^ Therefore, we speculated that the nano complex might promote axon growth by activating the mTOR signaling pathway. Confirmed using western blot experiment, the phosphorylation level of mTOR in the CM‐miR‐MSN and miR‐MSN group was significantly higher than that in the control group, suggesting that miRNA‐124‐3p promotes axon growth through activation of mTOR signaling pathway (Figure 2G,H). Meanwhile, Kras and P53 signaling pathways were up‐regulated. Although some studies have shown that the Kras gene can enhance the axon regeneration ability of retinal ganglion cells,^[^ ^43^ ^]^ few studies have dug into this and its relationship with axon growth still needs to be further verified. Similarly, literature has demonstrated the considerable potential of P53 in the nervous system,^[^ ^44^ ^]^ yet its role in promoting axon growth has not been conclusive. Therefore, verification of these two signaling pathways was not further pursued. In summary, after the uptake by neurons, the nano complex promotes axon growth mainly by activating mTOR signaling pathway.

### 2.4 Phagocytosis of the Nano Complexes by Microglia and Promotion of Polarization Toward M2 Phenotype

**Figure 3:**
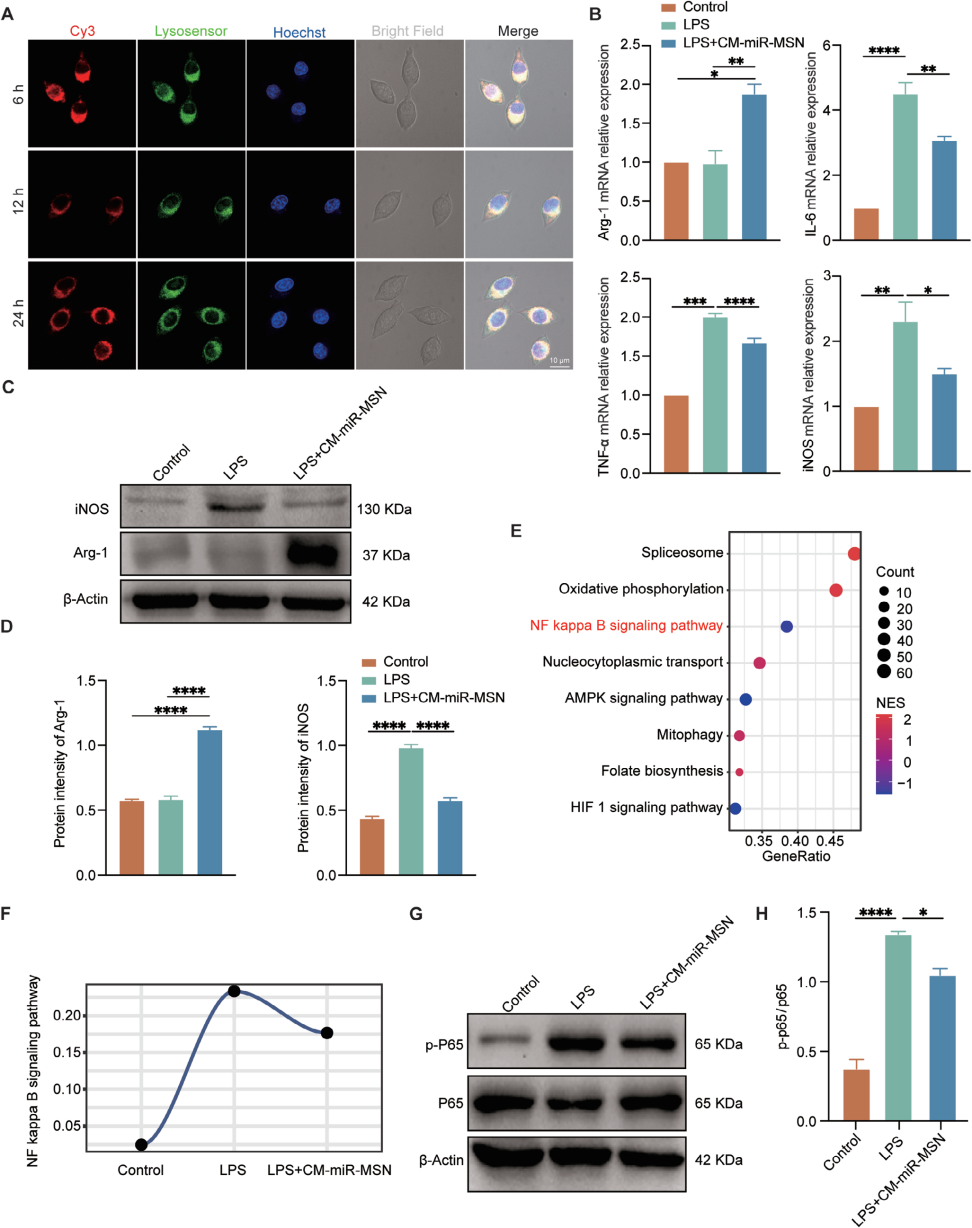
Phagocytosis of nano complexes by BV2 and promotion of BV2 polarization toward the M2 phenotype in vitro. A) Representative fluorescence images of CM‐miR‐MSN, lysosomes, and nuclei; bright‐field images and merged images of BV2 cultured with CM‐miR‐MSN for 6, 12, and 24 h. The miRNA was stained with Cy3 (red), lysosomes with Lysosensor (green), and nuclei with Hoechst (blue). Scale bar: 10 µm. B) The mRNA expression of Arg‐1, IL‐6, TNF‐α and iNOS was detected using quantitative polymerase chain reaction. Statistical analysis was performed using one‐way analysis of variance (ANOVA); *p < 0.05, **p < 0.01, ***p < 0.001, and ****p < 0.0001. C) The expression of iNOS and Arg‐1 after lipopolysaccharide (LPS) induction and CM‐miR‐MSN treatment. D) Quantitative analysis of (C). Statistical analysis was performed using one‐way ANOVA; *p < 0.05 and ***p < 0.001. E) Gene ratio analysis and F) NF‐κB signaling pathway expression expected by RNA‐seq. G) The expression of NF‐κB p‐P65 and P65 in each group was determined by western blot. H) Quantitative analysis of the p‐P65/P65 ratio. Statistical analysis was performed using one‐way ANOVA; *p < 0.05 and ****p < 0.0001.

Growing evidence suggests that microglia/macrophages are crucial cellular players in inflammatory events. The activated microglia/macrophages gather at the site of SCI and persist for weeks, promoting both injury and repair.^[^ ^45^ ^]^ These disparate effects are associated with different microglia/macrophage phenotypes, namely, “classically activated” pro‐inflammatory cells (M1) or “alternatively activated” anti‐inflammatory cells (M2).^[^ ^46^, ^47^ ^]^ The M2 phenotype plays an anti‐inflammatory and neuroprotective role through arginase‐1 (Arg‐1).^[^ ^48^ ^]^ Conversely, the M1 phenotype sustains the long‐term synthesis and release of divergent proinflammatory mediators, including proinflammatory cytokines interleukin‐6 (IL‐6), tumor necrosis factor‐α (TNF‐α), inducible nitric oxide synthase (iNOS),^[^ ^46^, ^49^ ^]^ and various chemokines. To assess the phagocytosis of miR‐MSN and CM‐miR‐MSN by BV2 cells, we co‐cultured them separately with BV2 cells for 6, 12, and 24 h. Cy3 signals highly coincided with lysosomes of BV2 cells in both groups after 6 h (**Figure** **3**A; Figure S5, Supporting Information), and the uptake capacities over the two composites appeared similar. This aligns with our speculation that BV2 has a strong capacity for engulfing, to which the neuron‐targeting CM contributes little. Next, we verified whether the nano complexes alter the microglia's polarization or whether miRNA‐124‐3p promotes M2 polarization. Lipopolysaccharide (LPS)‐primed BV2 cells were incubated with CM‐miR‐MSN to verify if the nano complexes induce anti‐inflammatory effects. LPS can trigger BV2 cells to polarize toward the M1 phenotype.^[^ ^50^ ^]^ After incubating for 24 h, we extracted BV2 total RNA for qPCR analysis. The results revealed that compared with the control group, the expression of M1 markers iNOS, IL‐6, and TNF‐α increased in the LPS‐primed group, but decreased after CM‐miR‐MSN treatment. Conversely, the M2 marker Arg‐1 expression was upregulated in the LPS + CM‐miR‐MSN group (Figure 3B). Similarly, the western blot also showed that the LPS group exhibited a significant increase in iNOS expression. In contrast, iNOS expression was lower, whereas Arg‐1 protein expression was higher in the LPS + CM‐miR‐MSN group than in the LPS group (Figure 3C,D). Next, we further verified that miRNA‐124‐3p could promote BV2 polarization toward the M2 phenotype. With iNOS/CD68 and Arg‐1/CD68 immunofluorescence intensity representing the rate of M1 and M2 in microglia, respectively, we found that treatment with CM‐miR‐MSN substantially reduced iNOS fluorescent intensity and increased Arg‐1 intensity (Figure S6, Supporting Information). These data provided evidence that the CM‐miR‐MSN can promote BV2 polarization toward the M2 phenotype.

To further investigate the mechanism of how miRNA‐124‐3p promotes BV2 polarization toward the M2 phenotype, we sequenced RNA from the control, LPS, and LPS + CM‐miR‐MSN groups. GSEA revealed significant differences in multiple signaling pathways between the LPS + CM‐miR‐MSN and LPS groups. Specifically, the spliceosome and oxidative phosphorylation pathways were activated, whereas the NF‐κB signaling pathway and other inflammation‐related pathways were suppressed (Figure 3E). Given the pivotal role of the NF‐κB signaling pathway in inflammation regulation,^[^ ^8^ ^]^ we further investigated its activation across the three groups. Intriguingly, a remarkable difference was observed between the LPS group exhibiting evident activation of the NF‐κB signaling pathway and the LPS + CM‐miR‐MSN group, which was suppressing it. This observation suggests that CM‐miR‐MSN exerts its anti‐inflammatory effects by modulating the NF‐κB signaling pathway (Figure 3F). Consistent with RNA‐seq, the western blot analysis also revealed higher phosphorylated levels of NF‐κB in the LPS group than in the control group, whereas it was completely suppressed in the presence of LPS + CM‐miR‐MSN. This experiment confirmed that miRNA‐124‐3p promotes BV2 cell polarization toward the M2 phenotype by suppressing the NF‐κB signaling pathway (Figure 3G,H).

### 2.5 Nano Complexes Target the Spinal Cord and Are Engulfed by Neurons and Microglia

**Figure 4:**
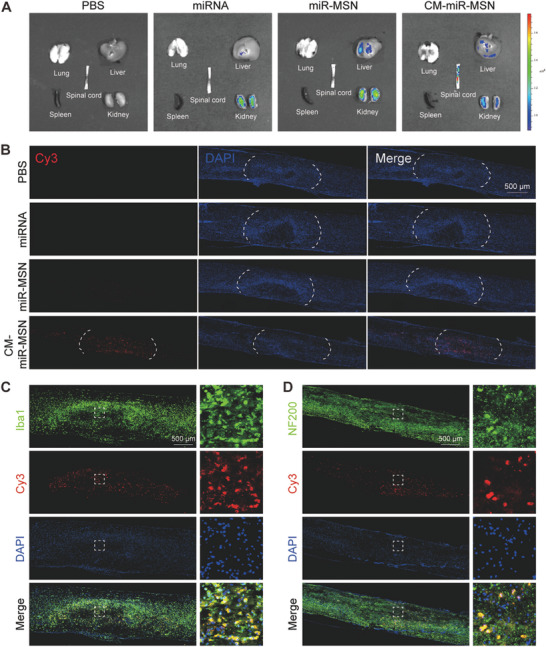
In vivo distribution of miRNA, miR‐MSN, and CM‐miR‐MSN after tail vein injection, miRNAs labeled with Cy3 (red). A) Distribution of fluorescence intensity in the major organs. B) Distribution of Cy3 fluorescence signal in the spinal cord injury region. Scale bar: 500 µm. C) Representative images showing the colocalization of Cy3‐labeled CM‐miR‐MSN and Iba1‐labeled microglia. Scale bar: 500 µm. D) Representative images showing the colocalization of Cy3‐labeled CM‐miR‐MSN and NF200‐labeled neurons. Scale bar: 500 µm.

The transiently disrupted BSCB after SCI allows for extravascular access of drugs from the bloodstream. However, a lack of specific drug binding sites in the injured area hinders passive drug retention, and it leads to subsequent washout over time, thereby considerably limiting the efficacy of systemic drug therapy.^[^ ^24^ ^]^ We investigated whether the specific binding of RVG with neuron nAChR can subsequently assist nano complex accumulation in the spinal cord. We adopted a contusive SCI mouse model that successfully mimicked the pathology of clinical SCI to evaluate the targeting ability of the nano complex for the spinal cord. The mice were randomly assigned into the PBS (control), miRNA‐124‐3p, miR‐MSN group, and CM‐miR‐MSN groups. The treatment solutions were injected into the tail vein at 100 µL after contusive SCI induction, with each mouse receiving 2.5 nmol miRNA‐124‐3p (Cy3‐labeled). The Cy3 fluorescent signals in major organs were visualized after 24 h through bioimaging studies to study the biodistribution of the nano complexes in vivo. In the PBS group, no fluorescent signals were detected in any major organs. In the miRNA and the miR‐MSN groups, fluorescent signals were primarily observed in the kidneys and liver, with a barely visible signal detected in the SCI region. In the CM‐miR‐MSN group, however, the SCI region showed a robust Cy3 signal, whereas the liver and kidneys exhibited significantly weaker fluorescent signals than those in the miR‐MSN group (**Figure** **4**A). Thus, modifying the CM with RVG facilitated the passive retention of the nano complexes at the SCI site.

Subsequently, we further confirmed findings by analyzing frozen sagittal sections of injured spinal cord specimens from each group and found that the Cy3 fluorescence signal was abundant in the SCI region in CM‐miR‐MSN–treated mice but not in the PBS, miRNA, or miR‐MSN group (Figure 4B). Collectively, these results indicate the excellent targeted specificity of the CM‐miR‐MSN toward the SCI region.

We performed immunofluorescence staining of spinal cord sections from the CM‐miR‐MSN group, specifically labeling neurons and microglia with NF200 and Iba1, respectively.^[^ ^8^ ^]^ As the result revealed, Cy3 fluorescent signals were co‐localized with neurons and microglia (Figure 4C,D). This finding was ascribed to the binding of RVG with nAChR, which allows a portion of the nano complexes to successfully remain at the SCI site via blood circulation and be taken up by neurons. However, since the nano complexes accumulated at the SCI site, another portion of the nano complexes will be phagocytosed by microglia/macrophages. Altogether, the above results suggest that with the aid of neuron‐targeting RVG, CM‐miR‐MSN crossing through the damaged BSCB could specifically target the injured spinal cord where neurons and microglia would take it up to deliver miRNA‐124‐3p for SCI repair.

### 2.6 Nano Complexes Promote Axon Regeneration and Microglia Polarization to M2

**Figure 5:**
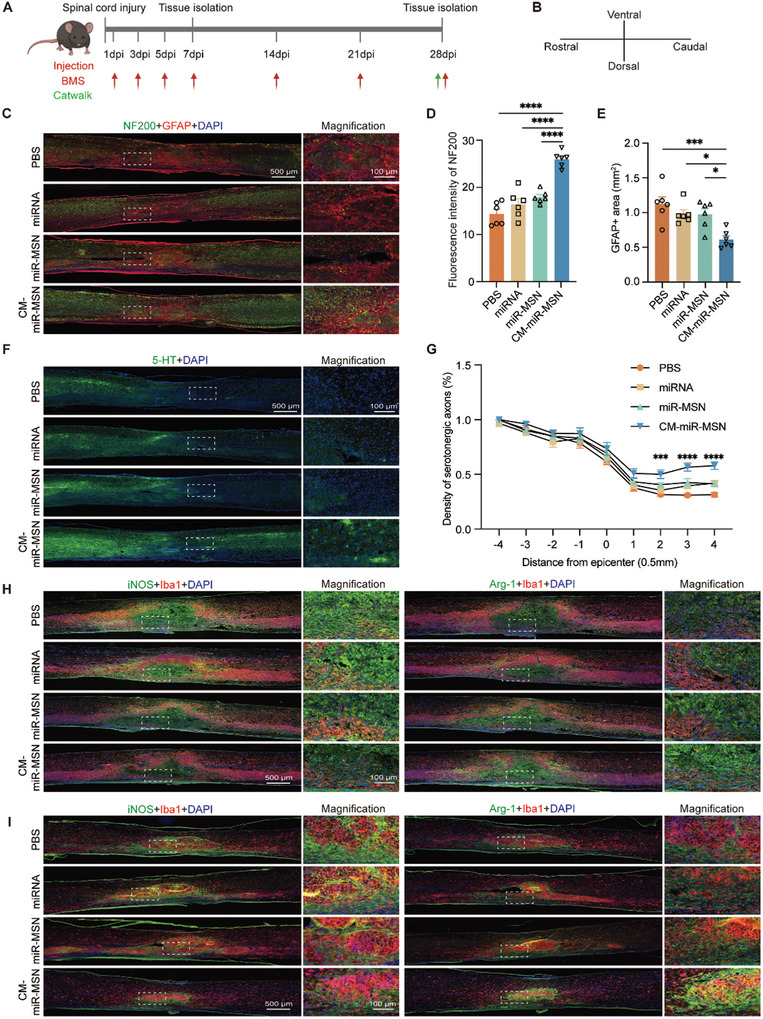
The nano complexes promote axon regeneration and microglia polarization to M2 in the contusive spinal cord injury (SCI) mouse model. A) The scheme of time axis shows the design of the animal study. B) Diagram for the layout direction of spinal cord section images. C) Immunostaining of NF200‐positive neuro‐filaments and GFAP‐positive astrocytes in sagittal sections of the injured area. Scale bar: 500 µm. D) Quantification of the NF200 fluorescence intensity and E) GFAP‐positive area in the injury center (PBS: n = 6; miRNA: n = 6, miR‐MSN: n = 6; CM‐miR‐MSN: n = 6. Statistical analysis was performed using one‐way analysis of variance [ANOVA]; *p < 0.05, ***p < 0.001, and ****p < 0.0001). F) Representative images of regenerating 5‐HT‐positive serotonergic axons beyond the lesion site. Scale bar: 500 µm. G) Quantification of regenerating serotonergic axons at the indicated distance beyond the lesion (PBS: n = 6; miRNA: n = 6; miR‐MSN: n = 6; CM‐miR‐MSN: n = 6; ***p < 0.001, and ****p < 0.0001, one‐way ANOVA). H) Representative sections from the same specimen in each group with immunolabeled iNOS and Arg‐1 at day 7 after SCI. Scale bar: 500 µm, inset: 100 µm. I) Representative sections from the same specimen in each group with immunolabeled iNOS and Arg‐1, respectively at day 28 after SCI. Scale bar: 500 µm, inset: 100 µm.

We evaluated the therapeutic effect of CM‐miR‐MSN in SCI treatment through a series of experiments in vivo, with a schedule detailed in **Figure** **5**A. Eight‐week‐old mice were randomly divided into the PBS, miRNA‐124‐3p, miR‐MSN, and CM‐miR‐MSN groups. All mice underwent contusive SCI induction and were subsequently injected with PBS, miRNA‐124‐3p, miR‐MSN, or CM‐miR‐MSN solution, respectively, at a dose of 100 µL. Animals were injected every other day during the first‐week post‐injury and once a week thereafter, with each mouse receiving 1.8 nmol of miRNA‐124‐3p per injection through the tail vein. We determined the mice's Basso Mouse Scale (BMS) score at each injection time point and used the CatWalk assay at 28 days post‐injury to evaluate locomotion recovery. To examine the polarization direction of microglia in early‐phase SCI, we collected spinal cord specimens from 3 mice in each group on day 7 post‐injury for sagittal sections. Blood was collected from 6 mice in each group on day 28 post‐injury for biocompatibility assessment and sent for blood chemistry analysis. Spinal cords, livers, spleens, lungs, and kidneys were harvested after transcardiac perfusion for hematoxylin & eosin (HE) staining and subsequent microscopic observation. On the same day, we collected spinal cord specimens from six mice from each group, and we made sagittal sections for immunofluorescence staining to observe axonal regrowth and microglia polarization in late‐phase SCI. All spinal cords in this study were collected at 5 mm anterior and posterior to the injury center.

We immunolabeled NF200, GFAP, and 5‐HT in the spinal cord sections collected at 28 days post‐injury to explore whether CM‐miR‐MSN promotes axon regeneration in the injury center. NF200 continuity was disrupted in the injury area due to axonal disruption after SCI, and it was thus adopted as an indicator for axon regeneration.^[^ ^8^, ^51^ ^]^ As suggested by the immunofluorescence staining, no significant difference was found between the miRNA‐124‐3p or miR‐MSN group and the PBS group. However, the CM‐miR‐MSN group showed a significant increase of NF200‐positive area (Figure 5B–D; Figure S5, Supporting Information). These data provide strong evidence that CM‐miR‐MSN can promote axon regrowth. Numerous GFAP‐positive astrocytes gather and form scars in the SCI center, creating an inhibitory environment that hinders axon regeneration.^[^ ^8^, ^45^ ^]^ We found that all groups displayed remarkable GFAP aggregation in the injury center. However, the GFAP‐positive area was significantly smaller in the CM‐miR‐MSN group than in the other three groups (Figure 5C,E; Figure S7, Supporting Information), indicating that CM‐miR‐MSN effectively inhibited scar formation by GFAP+ astrocytes, further providing a nurturing environment for axon regeneration.

Given that 5‐HT–positive axons are disrupted below the lesion site,^[^ ^9^, ^52^ ^]^ we immunolabeled these axons in sagittal sections of spinal cords collected from the lesion center. As expected, all SCI mice exhibited significant 5‐HT–positive axon disruption. Conversely, the CM‐miR‐MSN group displayed longer extensions of 5‐HTergic fibers and increased axon length (Figure 5F,G; Figure S8, Supporting Information). These findings implicated the strong potential of CM‐miR‐MSN in enabling axon regeneration.

Neuroinflammation plays a central role in secondary SCI. Microglia are the predominant innate immune cells of the central nervous system that rapidly activate and aggregate in the lesion center after an injury.^[^ ^53^ ^]^ With the integration of RVG, the nano complex we designed is expected to accumulate in the SCI center through blood circulation more readily, bind with nAChR, and be taken up by neurons. Generally, maximizing the cellular uptake of the nano complexes by neurons is most favorable for augmenting axonal regeneration. However, the nano complexes will inevitably be phagocytosed by microglia aggregating in the injury center. To effectively and thoroughly utilize the nano complex, we intend to testify that CM‐miR‐MSN promotes the polarization of microglia toward the anti‐inflammatory M2 phenotype, which makes the “constraint” microglial phagocytosis conducive to SCI repair. To investigate whether the nano complex stimulates spinal cord microglia polarization toward the M2 phenotype in early‐ and late‐phase SCI in vivo, we performed immunofluorescence staining on sagittal spinal cord sections collected on days 7 and 28 post‐injury. Significant aggregation of Iba1‐positive microglia was observed in all groups, indicating the persistent presence of microglia in the SCI center on days 7 and 28 post‐injury (Figures S9–S12, Supporting Information). Moreover, compared with the PBS group, the CM‐miR‐MSN group showed lower iNOS fluorescent intensity and increased Arg‐1 intensity in both early‐ and late‐phase SCI. However, the treatments with miRNA and miR‐MSN did not show any apparent effects (Figures S7–S10, Supporting Information). Statistical analysis calculated the ratio of Arg‐1/iNOS fluorescent intensity, which reflects the M2/M1 phenotype ratio in two neighboring sections from the same specimen. Intriguingly, whether on days 7 or 28 post‐injury, the treatment of CM‐miR‐MSN seemed to reverse the ratio obtained in the PBS group (Figure 5H,I; Figure S13, Supporting Information), demonstrating that CM‐miR‐MSN promoted the M2 polarization of microglia in the SCI center, which could greatly facilitate the rehabilitation of the inflammatory microenvironment in SCI and accelerate SCI repair.

The improvement in SCI can be evaluated by HE staining.^[^ ^54^ ^]^ Thus, we performed HE staining on sagittal spinal cord sections collected at 28 days post‐injury. The results showed that morphologically, CM‐miR‐MSN treatment significantly reduced the SCI center's lesion area, indicating a significant reparative effect on SCI (Figure S14, Supporting Information). The above results established that CM‐miR‐MSN successfully targeted the SCI center, where it enhanced axonal regeneration, regulated the local microenvironment at the injury site, and promoted microglia M2 polarization, thereby boosting SCI repair.

### 2.7 Nano Complexes Enhance Motor Function Recovery

**Figure 6:**
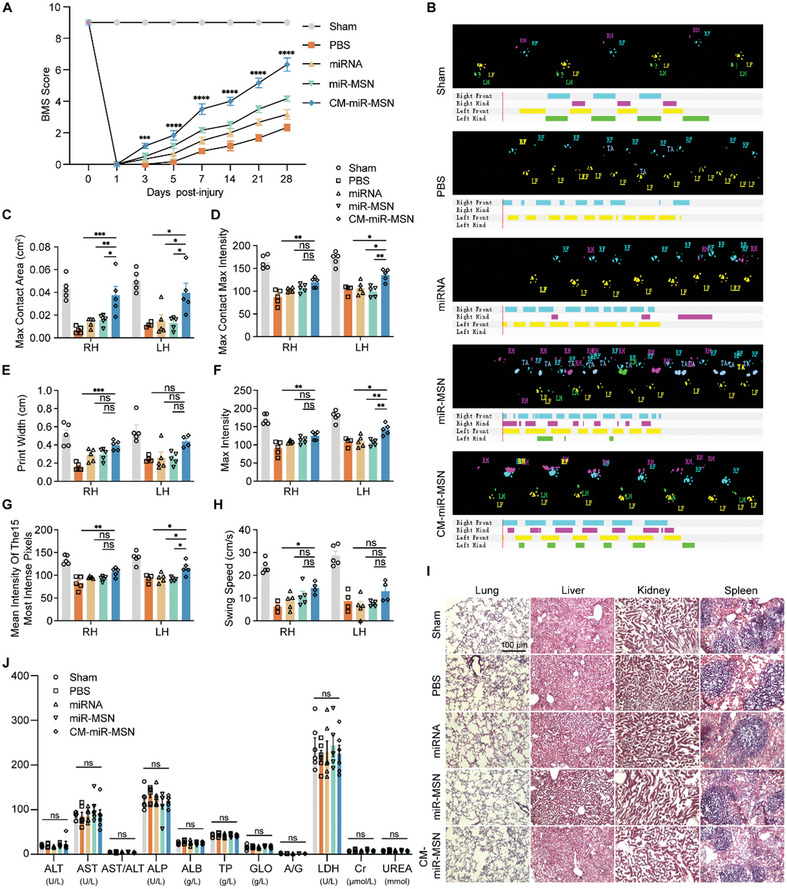
Functional recovery in contusive spinal cord injury (SCI) mice after treatment with miRNA, miR‐MSN, and CM‐miR‐MSN. A) Basso Mouse Scale score of SCI mice treated with PBS, miRNA, miR‐MSN, and CM‐miR‐MSN (Sham: n = 6; PBS: n = 6; miRNA: n = 6; miR‐MSN: n = 6; CM‐miR‐MSN: n = 6. Statistical analysis was performed using two‐way analysis of variance [ANOVA]; ***p < 0.001 and ****p < 0.0001). B–H) Locomotor recovery after treatment with PBS, miRNA, miR‐MSN, and CM‐miR‐MSN determined by CatWalk gait analysis (Sham: n = 5; PBS: n = 5; miRNA: n = 5; miR‐MSN: n = 5; CM‐miR‐MSN: n = 5; RH: right hindlimb; LH: left hindlimb; Statistical analysis was performed using one‐way ANOVA; *p < 0.05, **p < 0.01, and ***p < 0.001). B) Representative footprints after treatment. C) Max Contact Area for bilateral hindlimbs. D) Max Contact Max Intensity for bilateral hindlimbs. E) Print Width for bilateral hindlimbs. F) Max Intensity for bilateral hindlimbs. G) Mean Intensity of the 15 Most Intense Pixels for bilateral hindlimbs. H) Swing speed for bilateral hindlimbs. I) Hematoxylin and eosin staining of kidney, liver, lung, and spleen tissues from SCI mice treated with PBS, miRNA, miR‐MSN, or CM‐miR‐MSN by tail vein injection and sham surgery mice. Scale bar: 100 µm. J) Blood chemistry data obtained from mice after PBS, miRNA, miR‐MSN, and CM‐miR‐MSN treatment. Sham: n = 6; PBS: n = 6; miRNA: n = 6; miR‐MSN: n = 6; CM‐miR‐MSN: n = 6. Statistical analysis was performed using two‐way ANOVA, ns, not significant (p > 0.05).

Motor function recovery in the SCI mouse model can be evaluated using the BMS and gait analysis. Hindlimb movement was assessed using an open‐field test and BMS in all independent experiment groups.^[^ ^55^, ^56^, ^57^ ^]^ A day after the injury, the BMS scores of all animals declined to 0 (**Figure** **6**A). Subsequently, all groups of mice showed recovery trends, but to varying extents. On day 28 post‐injury, the PBS group obtained an average score of 2.33333, the miR group 3.16667, the miR‐MSN group 4.16667, and the CM‐miR‐MSN group 6.33333, the highest score. Thus, CM‐miR‐MSN significantly promoted motor recovery after SCI in mice (Figure 6A). We also employed CatWalk XT system, a footprint analysis system, for detailed and quantified evaluation of movements. This system records mouse gaits as they walk across an illuminated floor. As suggested by the results, more coordinated forelimb and hindlimb movement was observed in CM‐miR‐MSN–treated mice compared with the PBS group (Figure 6B; Videos [Link], [Link], [Link], [Link], [Link], Supporting Information). Intriguingly, the swing duration of both forelimbs and hindlimbs decreased after contusive SCI but increased again after CM‐miR‐MSN treatment, especially in the hindlimbs. Furthermore, treatment with CM‐miR‐MSN significantly improved the outcomes of multiple parameters of functional recovery, including maximum contact area, max contact, max intensity, print width, max intensity, mean intensity of the 15 most intense pixels, and swing speed (Figure 6C–H).

To verify the biocompatibility of the nano complex in vivo, we preserved lungs, livers, kidneys, spleens, and serum from the sham, PBS, miRNA‐124‐3p, miR‐MSN, and CM‐miR‐MSN groups at 28 days post‐injury. Lung, liver, and spleen tissues were sectioned and stained with HE for analysis, which revealed no observable abnormal signs in all experiment groups in comparison with the PBS group or sham group, thereby demonstrating the excellent biocompatibility of the nano complex (Figure 6I). Blood chemistry did not indicate any drug‐related liver impairment since all groups exhibited normal levels of ALT, AST, and ALP (Figure 6J). Cr and UREA, indicators of renal function, were at normal levels, suggesting no detectable nephrotoxicity. Hematological analysis revealed that blood parameters, such as albumin, globulin, and LDH, were within the normal range in the treated groups, indicating no inflammation triggered by the nano complexes. The above results prove that the nano complex enhances motor function restoration after SCI and is free of inducing organ toxicity or inflammatory responses in vivo.

## 3 Conclusion

We developed an RVG‐assembled HucMSCM‐camouflaged nano complex with a miRNA‐124‐3p–loaded MSN core for targeted repair of SCI. Our research unequivocally demonstrated three distinct merits of this nano complex in SCI repair: i) sufficient miRNA release at the injured spinal cord because of adequate passage through the BSCB; ii) multifaceted effects, such as enhancing axonal outgrowth and regulating the local microenvironment; and iii) prevention of secondary spinal cord damage via an intravenous mode of administration instead of localized spinal cord administration. Although our study explicitly demonstrated the efficacy and safety of CM‐miR‐MSN in a contusive SCI mouse model, it has some limitations. Specifically, the degradability of the nano complex in vivo was not directly elucidated. To summarize, we developed a new nano complex targeting SCI sites to stimulate axonal growth, regulate the local microenvironment, and accelerate SCI repair. This study offers a nano complex–based strategy for treating SCI in a site‐specific, noninvasive manner and provides insights into various applications of functional miRNAs in SCI.

## 4 Experimental Section

### Culture of HucMSCs, RVG Transfection, and Isolation of Cell Membrane

Human umbilical cords were obtained from full‐term cesarean section surgeries at Shandong University Qilu Hospital (Jinan, China). The patients were informed beforehand and provided consent for donation. All experimental procedures involving human tissues were approved by the Research Ethics Committee of Qilu Hospital, Shandong University (Jinan, KYLL‐202308‐032). The MSCs were obtained by tissue mass culture and cultured in specialty media (BasalMedia, T310KJ, Shanghai, China). The lentivirus (sp‐RVG‐GFP‐tm) (Genechem, Shanghai, China) was added when the cells were 50% confluent. At 90% confluency, the cells were screened with puromycin and observed under a fluorescent microscope. The cells to the third or fourth generation until fluorescent microscopic inspection suggested the GFP‐expressing HucMSCs accounted for 95% was passaged. When the culture reached 90% confluency, 0.25% trypsin‐EDTA was added to the cells, and cells were centrifuged at 1000 × g for 5 min. The cells were then suspended in a hypotonic solution containing 20 mM Tris‐HCl (pH 7.5), 10 mM KCl, 2 mM MgCl2, and 1 mM phenylmethylsulfonyl fluoride (Solarbio, Beijing, China) at 4 °C followed by treatment with an ultrasound processor (Scientz‐IID, China). After being centrifuged at 2000 × g for 30 min at 4 °C, the supernatant was collected and centrifuged again at 15 000 × g for 30 min at 4 °C to obtain CM. The isolated CM was preserved at −80 °C until further use.^[^ ^23^ ^]^

### Preparation and Characterization of the Nano Complexes

CM‐MSN was synthesized by cloaking CM to MSN via thermodynamic forces. First, the CM with a protein concentration of 0.2 mg mL^−1^ was added to animated MSN dispersion purchased from XFNANO (103 828, Jiangsu, China). The resulting dispersion was processed in an ultrasonic bath at 42 kHz for 2 min and centrifuged at 5000 × g for 30 min at 4 °C to prepare the CM‐MSN. The synthesis of CM‐miR‐MSN was accomplished in three steps. First, miR‐124‐3p (Sangon, Shanghai, China) to the MSN dispersion at a weight ratio of 1:20 and let the mixture stand for 10 min at 37 °C to enable sufficient miRNA loading was added. Second, the CM with 0.2 mg mL^−1^ protein to the dispersion was added. The mixture was processed in an ultrasonic bath at 42 kHz for 2 min and then centrifuged at 5000 × g for 30 min at 4 °C to obtain the CM‐miR‐MSN. The particle size and surface charge of the MSN and CM‐MSN were measured in a zetasizer (DLS) (Malvern Zetasizer, Nano ZS90, UK). The MSN and CM‐MSN were suspended in PBS (pH 7.4) at 0.1 mg mL^−1^. All measurements were performed three times at room temperature. TEM (Nippon Electron Corporation, JEM2100, Japan) was used for morphological imaging of MSN, 12 h FBS‐treated MSN, 24 h FBS‐treated MSN, CM‐MSN, CM‐miR‐MSN, and elemental mapping of CM‐MSN.^[^ ^23^ ^]^ For release studies, miR‐MSN and CM‐miR‐MSN were placed separately in Eppendorf tubes and then incubated at 37 °C in 1 mL of solution with pH = 4.5 or 7.0. The Eppendorf tubes were centrifuged at 14 000 rpm for 10 min at every fixed time point. The supernatant was collected and replaced with a fresh solution. The amount of miRNA in the supernatant was measured using an ultramicro spectrophotometer (Micro Drop, BIO‐DL, Shanghai, China) for four consecutive days. All release studies were conducted in triplicate.

### Acquisition and Culture of Cortical Primary Neurons

Primary neurons were obtained from the embryo cortex of a C57BL/6 mouse (Vital River Laboratory Animal Technology, Jinan, China).^[^ ^58^, ^59^ ^]^ All the animal‐involved experiments received approval from the Ethics Committee of Shandong University (23 035, Jinan, Shandong, China). Pregnant mice were sacrificed at day 16 of gestation and sterilized with 75% pre‐cooled ethanol. The brain cortices were harvested under a stereomicroscope (Chongqing Optec Instrument Co., Ltd., Chongqing, China) and cut into pieces of ≈1 mm^3^ in size. Next, these were digested in the mixture of 0.2% (w/v) papain (LS003119, Worthington Biochem, Lakewood, NJ, USA) and 0.004% (w/v) DNase solution (Sigma–Aldrich, St. Louis, MO, USA) at 37 °C for 20 min until the enzyme became fully inactivated, during which the tissue underwent gentle pipetting 8–10 times every 5 min. Subsequently, the tissue was centrifuged at 200 × g for 5 min. Cell clumps were removed by delicately aspirating and filtering the supernatant through a 40‐µm cell strainer (SPL Life Sciences). The cells were suspended in Dulbecco's Modified Eagle's Medium (Invitrogen), 10% fetal bovine plasma (Cellmax), and 1% penicillin‐streptomycin (Gibco). The cells were counted and seeded into appropriate wells coated with poly‐D‐lysine (P4707, Sigma‐Aldrich). The medium was substituted with neuronal medium (Neurobasal, 2% B27 supplement, Invitrogen; 1 mM l‐glutamine, Invitrogen; and 1% penicillin and streptomycin, Invitrogen) 4 h later, half of the neuronal medium being replaced every 3 days ever since.

### CCK‐8 Assays

Cytotoxicity of miR‐MSN and CM‐miR‐MSN was assessed using a CCK‐8 kit (C0037, Beyotime, Shanghai, China). Primary neurons were plated on a 96‐well plate at 1 × 10^5^/well. Three days after harvesting primary neurons, the neuronal medium was substituted with mixtures of neuronal medium and PBS, miR‐MSN or CM‐miR‐MSN for 100 µL for 24, 48, and 72 h. Next, 10 µL of CCK‐8 solution was added to the well. After a 4 h incubation, the absorbance at 450 nm wavelength was measured for each well using a microplate reader. BV2 cells purchased from Procell (PM150410, Wuhan, China) were plated on a 96‐well plate at 5 × 10^3^/well. After cells were attached, 100 µL medium (Dulbecco's Modified Eagle's Medium, Invitrogen was added; 10% fetal bovine plasma, Cellmax; and 1% penicillin‐streptomycin, Gibco), which contained PBS, LPS, or LPS + CM‐miR‐MSN, respectively. After another 24 h, 10 µL CCK‐8 solution was added, and the mixtures were incubated for 1 h, after which the absorbance at 450 nm wavelength was measured.

### Live/Dead Assay

Primary neurons were plated on a 24‐well plate at 2 × 10^5^/well. Medium containing PBS, miR‐MSN, or CM‐miR‐MSN was added to the cells, which were cultured for 72 h. Meanwhile, BV2 cells were plated on a 24‐well plate at 5 × 10^4^/well, LPS or LPS + CM‐miR‐MSN was added, and the cells were cultured for 24 h. Subsequently, cells were stained with calcein and propium iodide PI (C2015M, Beyotime), and finally, the proportions of living (calcein+) and dead (PI+) cells was counted.

### Observation of Cellular Uptake

The cellular uptake in primary neurons and BV2 cells with a laser scanning confocal microscope (Carl Zeiss LSM 980, Oberkochen, Germany) was observed and captured. The primary neurons or BV2 cells were seeded to a glass‐bottomed cell culture dish at 5 × 10^4^ /well. After treatment with miR‐MSN or CM‐miR‐MSN with a final concentration of 100 nM miRNA (Cy3‐labeled), cells were cultured for another 6, 12, and 24 h. Subsequently, the cells were stained with Lysosensor (L7545, Thermo Fisher, Massachusetts, USA) and Hoechst (H1398, Thermo Fisher) for 15 min and observed under the LSM 980 microscope.

### Axon Guidance Spot Assays

CSPG was prepared as per the description in previous studies.^[^ ^9^, ^60^ ^]^ CSPG (2.5 µg mL^−1^; Millipore, CC117, MA, USA) of 5 µL was spotted onto a circle microscope glass coverslip coated with poly‐L‐lysine (P4707, Sigma–Aldrich) and Texas Red (Thermo Fisher, T7471, Massachusetts, USA) in the center to visualize the interface. After the spots dried, isolated primary cortical neurons were distributed at a density of 8 × 10^4^ cells on the coverslip. Cells were incubated simultaneously with PBS, miR‐MSN, or CM‐miR‐MSN after adhesion. Three days later, cells were fixed with tissue cell fixative solution and stained with anti‐β‐III‐tubulin primary antibody (1:200, ab18207, Abcam) and the corresponding secondary antibody. Imaging was conducted on an Olympus FV1000 inverted confocal microscope.

### CSPG‐Mediated Inhibition Tests

A 24‐well plate was coated with poly‐L‐lysine overnight at room temperature. CSPG (2.5 µg mL^−1^) was selectively added to the plates, and the culture dish was placed at 37 °C for 6 h. The obtained primary cortical neurons were seeded into culture plates at a density of 8 × 10^4^ cells per plate. Following adhesion, cells were incubated with PBS, miR‐MSN, or CM‐miR‐MSN for 3 days. The cells were fixed using a fixative solution, followed by staining with an anti‐βIII‐tubulin primary antibody (1:200, Abcam) and the appropriate secondary antibody. Imaging was conducted using the Olympus FV1000 inverted microscope, and axon length was measured using the ImageJ program.^[^ ^39^ ^]^

### Library Preparation and Sequencing

1 µg RNA per sample was collected for RNA sample preparation. Generation of sequencing libraries employed the NEBNext UltraTM RNA Library Prep Kit for Illumina (NEB, USA) following the manufacturer's guidelines. Index codes were incorporated to assign sequences to individual samples. The library quality was evaluated using the Agilent Bioanalyzer 2100 system. Subsequently, the libraries were sequenced in the Illumina Novaseq platform, generating 150‐bp paired‐end reads.

### Analysis of RNA‐seq Data

The raw data (raw reads) in fastq format underwent initial processing using custom Perl scripts to obtain high‐quality clean data (clean reads). Subsequent downstream analyses were conducted exclusively using the clean data. The paired‐end clean reads were aligned to the reference genome using Hisat2 (version 2.0.5). FeatureCounts, a component of Subread (version 2.0.4), was employed to quantify the number of reads mapped to each gene. The FPKM (Fragments Per Kilobase of transcript per Million mapped reads) value for each gene according to the gene length and number of reads mapped to that gene was subsequently calculated. Differential expression analysis comparing two conditions was performed using the DESeq2 R package (version 1.4.5). The differential expression of genes was determined using a model based on the negative binomial distribution. The resulting P‐values were adjusted with the Benjamini–Hochberg method. For GSEA, gene sets were accquired from the MSigDB Database (https://www.gsea‐msigdb.org/gsea/msigdb) and Reactome Pathway Database (https://reactome.org/). All expressed genes were ranked in advance based on their logFC values. GSEA analysis was conducted using the clusterProfiler package (version 4.8.1). Furthermore, gene set variation analysis (GSVA) to identify pathway changes using the GSVA R package (version 1.48.1) was performed.

### Western Blot Analysis

The membrane protein from HucMSCs was extracted using a membrane protein extraction kit (P0033, Beyotime, Shanghai, China), while the proteins from primary neurons and BV2 cells were collected from cell lysates. The protein samples were separated by sodium dodecyl sulfate–polyacrylamide gel electrophoresis and transferred onto a polyvinylidene fluoride membrane (Millipore). Subsequently, the membrane was blocked with 5% bovine serum albumin (BSA) in Tris‐buffered saline containing Tween20, after which the membrane was incubated with primary antibodies overnight at 4 °C and then horseradish peroxidase‐binding secondary antibody for 1 h at room temperature. The primary antibodies used were rabbit anti‐GFP antibody (1:1000, #2956, Cell Signaling Technology, Boston, MA, USA); rabbit anti‐Arg‐1 antibody (1:1000; 16001‐1‐AP, Proteintech, Chicago, USA); rabbit anti‐iNOS antibody (1:1000; 22226‐1‐AP, Proteintech); HRP‐conjugated β‐Actin monoclonal antibody (1:2000; HRP‐60008, Proteintech); rabbit anti‐mTOR (phospho) antibody (1:1000, ab109268, Abcam); rabbit anti‐mTOR antibody (1:1000, ab32028, Abcam); rabbit anti‐NF‐κB‐p65 (phospho) antibody; (1:1000, ab76302, Abcam), and rabbit anti‐NF‐κB‐p65 antibody (1:1000, ab32536, Abcam). The secondary antibody used was goat anti‐rabbit IgG‐HRP antibody (1:15 000, ab288151, Abcam). Then, an enhanced chemiluminescent substrate (Millipore) was applied to the blot for exposure, and the chemiluminescent signals were captured with a chemiluminescent imaging system (Bio‐Rad, California, USA).

### qPCR Analysis

The total RNA of BV2 cells was extracted with Trizol (Ambion, USA). 500 ng of total RNA was reversely transcribed to cDNA using a Taqman mRNA reverse transcription kit (Accurate Biology, Changsha, China). Real‐time qPCR was performed using a Light Cyder480 system (Roche, USA) with a 20 µL SYBR Green reaction system (Thermo Fisher, K1622, Massachusetts, USA). PCR amplification was performed for 40 cycles. It was normalized the expression of iNOS, Arg‐1, TNF‐α, and IL‐6 mRNA using GAPDH as the reference.

### Mouse Model of Spinal Cord Injury

This study employs female C57BL/6 mice aged 8–10 weeks old procured from Vital River Laboratory Animal Technology Co., Ltd. (Jinan, China) (Approval number: SCXK(Jing)2021 – 0006). Animals were kept in a 12‐h/12‐h light–dark cycle at 20–25 °C and 40%–60% humidity. Isoflurane (RWD, R510‐22, Shenzhen, China) for animal anesthetization was used. The skin, fascia and muscles around the 9th thoracic vertebra (T9) were cut longitudinally to expose the T8–T10 spinous processes. With the T8 and T10 spinous processes fixed by spinous process fixators, T9 laminectomy was performed to expose the spinal cord at the corresponding level fully. The contusion was accomplished utilizing an NYU Impactor‐III (WM Keck, USA) (5 g × 12.5 mm). Postoperatively, the mice were intramuscularly injected with cefuroxime sodium (6 mg mL^−1^, dissolved in saline; Hongtu, Nanjing, China) to prevent urinary tract infection, and bladders were squeezed twice a day to avoid urine retention.^[^ ^9^ ^]^

### In Vivo Distribution Assays

Four randomly assigned groups were separately injected PBS, Cy3‐marked miRNA, Cy3‐marked miR‐MSN, or Cy3‐marked CM‐miR‐MSN via the tail vein, with each mouse receiving 2.5 nmol miRNA in 100 µL solution. Mice were sacrificed 24 h after injection. Thereafter, images were captured, and Cy3 fluorescent signals were analyzed using the German Berthold Night Owl LB 983 NC100 imaging system. Next, the uptake of CM‐miR‐MSN by neurons and microglia in the spinal cord was analyzed. Spinal cord tissue was collected, fixed in 4% paraformaldehyde, dehydrated in sucrose, and embedded in an optimal cutting temperature (OCT) compound (Sakura, 4583, Japan). Specimens were cut into 10‐µm thick sections and detected with rabbit anti‐NF200 (1:2000, ab8135, Abcam) and mouse anti‐Iba1 (1:500, ab283319, Abcam). Polyclonal antibodies were then detected using goat anti‐rabbit IgG Alexa Fluor 488 (1:1000, ab150077, Abcam) and goat anti‐mouse 488 IgG Alexa Fluor 488 (1:1000, ab150113, Abcam). The landscape imaging system (OLYMPUS, 1900494S‐2, Japan) showed the colocalization of CM‐miR‐MSN (Cy3‐labeled) to different cell types.

### Transcardial Perfusion and Tissue Sectioning

After anesthesia, the mouse thorax and abdomen were opened from the xiphoid process along both costal margins. Pre‐cooled PBS was continuously transcardially perfused until the effluent from the right auricle appeared transparent, and the liver paled. Subsequently, pre‐cooled 4% paraformaldehyde was also transcardially perfused, followed by spinal cord harvesting. The latter was fixed in 4% paraformaldehyde overnight at 4 °C, dehydrated in sucrose solution, frozen in OCT, and sectioned at a thickness of 10 µm using a cryotome (Leica, CM3050S, Germany).

### Immunohistochemistry and Histology

Sections blocked with 5% normal goat plasma and 0.1% BSA in PBS, adding 0.1% Triton X‐100 in the blocking buffer. Afterward, sections were incubated with primary antibodies diluted in the blocking buffer overnight at 4 °C. The primary antibodies were rabbit anti‐Tuj1 (1:200, #3450, Cell Signaling Technology); anti‐NF200 (1:2000, ab8135, Abcam), anti‐5‐HT (1:5000, 20 080, Immunostar, Hudson, Wisconsin, USA); anti‐Arg‐1 (1:200; 16001‐1‐AP, Proteintech), anti‐iNOS (1:500; 22226‐1‐AP, Proteintech) mouse anti‐Iba1 (1:500, ab283319, Abcam); anti‐CD68 (1:50; ab955, Abcam); and anti‐GFAP (1:300, #3670S, Cell Signaling Technology). Next, the sections were incubated with the corresponding secondary antibody conjugated to Alexa Fluor 488 or 555 (1:500, Invitrogen, Carlsbad, California, USA) for 2 h at room temperature. Finally, the sections with PBS and covered them with a reagent to preserve fluorescence (ZLI‐9557, OriGene, Wuxi, China) was washed. Images were acquired using a scanning confocal laser microscope (OLYMPUS, 1900494S‐2, Japan). Quantification of GFAP–, NF200–, and 5‐HT–positive areas were performed via Image J software. Elimination of all backgrounds from each section while preserving the patterns of GFAP, NF200, and 5‐HT expression was achieved via threshold analysis. It was quantified axons labeled by tract tracing using NF200 or 5‐HT using the Image J software according to the manufacturer's protocol.

### In Vivo Biocompatibility in Major Organs

It was sacrificed mice at 28 days post‐injury to evaluate the biocompatibility of the nano complex. For microscopic observation, frozen sections of livers, spleens, lungs, and kidneys with Hematoxylin and eosin (H&E) (Biosharp, Hefei, China) was stained.

### Blood Chemistry

It was collected mouse blood before the transcardiac perfusion. Afterward, the blood samples were left to stand for 30 min and then centrifuged at 1500 rpm for 3 min. The serum was isolated and stored at −80 °C. Serum alkaline phosphatase (ALP), albumin (ALB), globulin (GLO), total protein (TP), glutamic aminotransferase (ALT), aspartate aminotransferase (AST), lactate dehydrogenase (LDH), creatinine (Cr), and UREA were assayed and analyzed by a clinical laboratory (Qilu Hospital, Shandong University).

### BMS for Locomotion

Mice were placed on a flat, enclosed field of 100 cm in diameter. The locomotion assessment lasts 4 min and involves two trained observers to evaluate hind limb joint movement, weight support, plantar stepping, and coordination.^[^ ^53^ ^]^ The score ranges from 0 to 9, with 0 representing complete paralysis and 9 representing normal function. Mice underwent this evaluation on day 1 post‐injury and weekly thereafter for 4 weeks.

### Gait Analysis on CatWalk

Mouse gait and locomotion were evaluated on the CatWalk XT system and CatWalk XT software (version 10.6, Noldus, Wageningen, the Netherlands).^[^ ^61^ ^]^ The system incorporates a corridor to direct movement, a glass walkway, and a high‐speed color camera. Each mouse underwent a training protocol on the walkway thrice before official analysis. The digital video camera set up beneath the glass captured the footprints of each mouse, which were analyzed using the CatWalk XT software. The parameters for evaluation include maximum contact area, max contact max intensity, print width, mean intensity, mean intensity of the 15 most intense pixels, and swing speed. The max contact area is the largest paw area that comes into contact with the glass plate. The max contact max intensity was the maximum intensity at max contact of the paw ranging from 0 to 255. The print width was the complete paw prints width (vertical direction). The mean intensity was the mean intensity of the whole paw. The mean intensity of the 15 most intense pixels was the mean intensity of the 15 pixels of the paw with the highest intensities. The swing speed was the paw's speed (distance unit/second) of the paw during swing.

### Statistical Analysis

All data were expressed as mean ± standard error of the mean. It was assessed statistical differences between treated groups with Sigma Stat (Systat Software Inc., Chicago, IL, US). As described in figure legends, both parametric and non‐parametric analyses were performed. The sample size was determined via G*Power 3.1.7 (Power analysis and sample size), and significance was determined by p < 0.05.

## Conflict of Interest

The authors declare no conflict of interest.

## Author Contributions

X.F. and L.S. contributed equally to this work. X.F., L.S., X.K., H.L., H.Z. and S.F. conceived the project and designed the experiments. X.F. and L.S. performed most of the cellular, biochemical, and animal experiments. Y.L., L.C., M.L., and T.Z. partially contributed to the biochemical experiments. Z.Y., W.L., Y.S., and J.L. partially contributed to the animal experiments. C.Z., B.B., E.E.‐Y.Y., Z.Q., P.X., J.Q., F.Y., and N.R. provided the technological supports for biochemical and animal experiments. X.K., H.L., H.Z., and S.F. edited the manuscript. S.F. and H.Z. provided financial support. All authors approved the final manuscript.

## Supporting information

Supporting Information

Supplemental Video 1

Supplemental Video 2

Supplemental Video 3

Supplemental Video 4

Supplemental Video 5

## Data Availability

The data that support the findings of this study are available from the corresponding author upon reasonable request.
