# Supplementary material for: Targeted Repair of Spinal Cord Injury Based on miRNA‐124‐3p–Loaded Mesoporous Silica Camouflaged by Stem Cell Membrane Modified with Rabies Virus Glycoprotein
Source: Adv Sci (Weinh). 2024 Mar 21;11(21):2309305. doi: 10.1002/advs.202309305 (PMC11151008; doi:10.1002/advs.202309305)
Supplement: Supplementary file 1 — Supporting Information [file ADVS-11-2309305-s003.pdf]

ADVANCED SCIENCE
Open Access

Supporting Information

for *Adv. Sci.*, DOI 10.1002/advs.202309305

Targeted Repair of Spinal Cord Injury Based on miRNA-124-3p–Loaded Mesoporous Silica Camouflaged by Stem Cell Membrane Modified with Rabies Virus Glycoprotein

Xiangchuang Fan, Lusen Shi, Zimeng Yang, Yiwei Li, Chi Zhang, Baoshuai Bai, Lu Chen, Elzat Elham-Yilizati Yilihamu, Zhangyang Qi, Wenxiang Li, Peng Xiao, Mingshan Liu, Jichuan Qiu, Fan Yang, Ning Ran, Yifan Shang, Jiaxing Liu, Tehan Zhang, Xiaohong Kong, Hong Liu*, Hengxing Zhou* and Shiqing Feng**

Supporting Information

Targeted Repair of Spinal Cord Injury Based on miRNA-124-3p–Loaded Mesoporous Silica Camouflaged by Stem Cell Membrane Modified with Rabies Virus Glycoprotein

Xiangchuang Fan, Lusen Shi, Zimeng Yang, Yiwei Li, Chi Zhang, Baoshuai Bai, Lu Chen, Elzat Elham-Yilizati Yilihamu, Zhangyang Qi, Wenxiang Li, Peng Xiao, Mingshan Liu, Jichuan Qiu, Fan Yang, Ning Ran, Yifan Shang, Jiaxing Liu, Tehan Zhang, Xiaohong Kong, Hong Liu*, Hengxing Zhou* and Shiqing Feng**

X. Fan, L. Shi, Z. Yang, C. Zhang, B. Bai, L. Chen, E. Elham-Y. Yilihamu, Z. Qi, M. Liu, N. Ran, Y. Shang, J. Liu, Prof. H. Zhou, Prof. S. Feng
Department of Orthopaedics, Qilu Hospital of Shandong University, Shandong University Centre for Orthopaedics, Cheeloo College of Medicine
Shandong University
Jinan 250012, P. R. China
E-mail: X. Kong: kongxh@sdu.edu.cn.; H. Zhou: zhouhengxing@sdu.edu.cn.; S. Feng: shiqingfeng@sdu.edu.cn.
N. Ran, T. Zhang, Prof. S. Feng
The Second Hospital of Shandong University, Cheeloo College of Medicine
Shandong University
Jinan 250033, P. R. China
E-mail: S. Feng: shiqingfeng@sdu.edu.cn.
Y. Li, J. Qiu, Prof. H. Liu
State Key Laboratory of Crystal Materials
Shandong University
Jinan 250100, P. R. China
E-mail: H. Liu: hongliu@sdu.edu.cn.
Prof. P. Xiao, Prof. F. Yang
Key Laboratory Experimental Teratology of the Ministry of Education, Department of Biochemistry and Molecular Biology, School of Basic Medical Sciences, Cheeloo College of Medicine
Shandong University
Jinan 250012, P. R. China
Prof. F. Yang, Prof. X. Kong, Prof. H. Zhou, Prof. S. Feng

Advanced Medical Research Institute
Shandong University
Jinan 250012, P. R. China
Prof. H. Liu
Jinan Institute of Quantum Technology, Jinan Branch, Hefei National Laboratory
Jinan, 250101, P. R. China.
Prof. S. Feng
Department of Orthopaedics, Tianjin Medical University General Hospital, International Science and Technology Cooperation Base of Spinal Cord Injury, Tianjin Key Laboratory of Spine and Spinal Cord
Tianjin Medical University
Tianjin 300052, P.R. China

* Corresponding Author:

Keywords: *spinal cord injury, axonal regeneration, targeted repair, microRNA-124-3p*

Figures

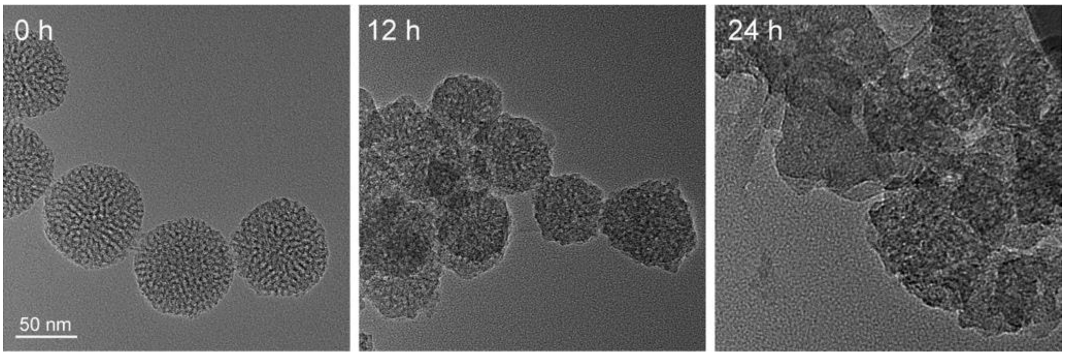

Figure S1. Transmission electron microscopy images of mesoporous silica nanoparticles before (0 h) and after incubation in fetal bovine serum for 12 and 24 h. Scale bar: 50 nm.

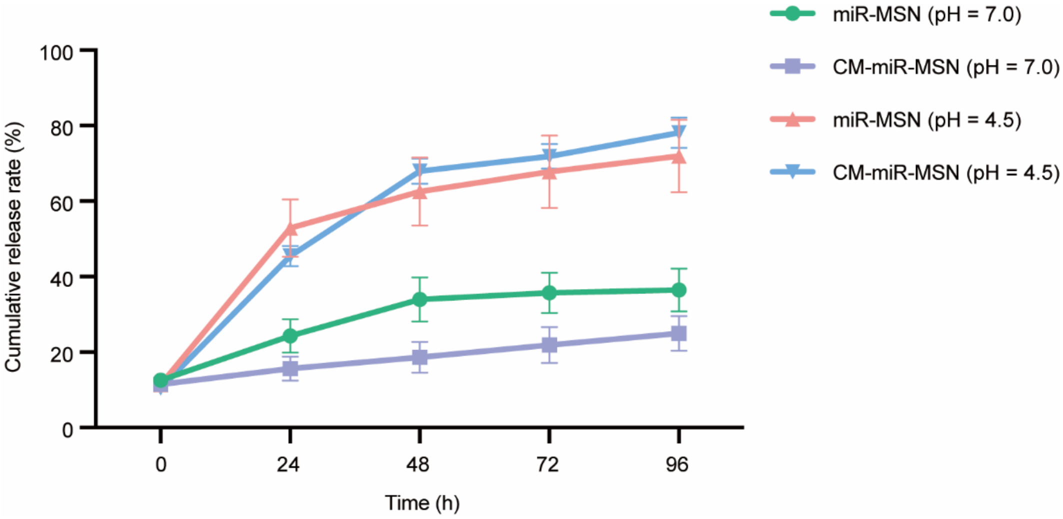

Figure S2. miRNA release curve of miR-MSN and CM-miR-MSN in a pH = 4.5 and pH = 7.0 environment.

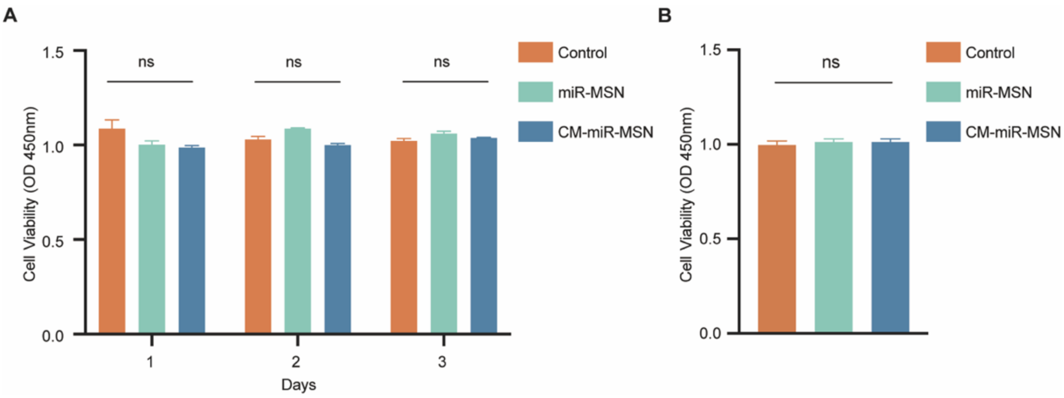

Figure S3. Cell viability of neurons (A) and BV2 (B) quantified by Cell Counting Kit-8 assay. We performed statistical analysis using one-way analysis of variance; ns, not significant ($p > 0.05$; n=3).

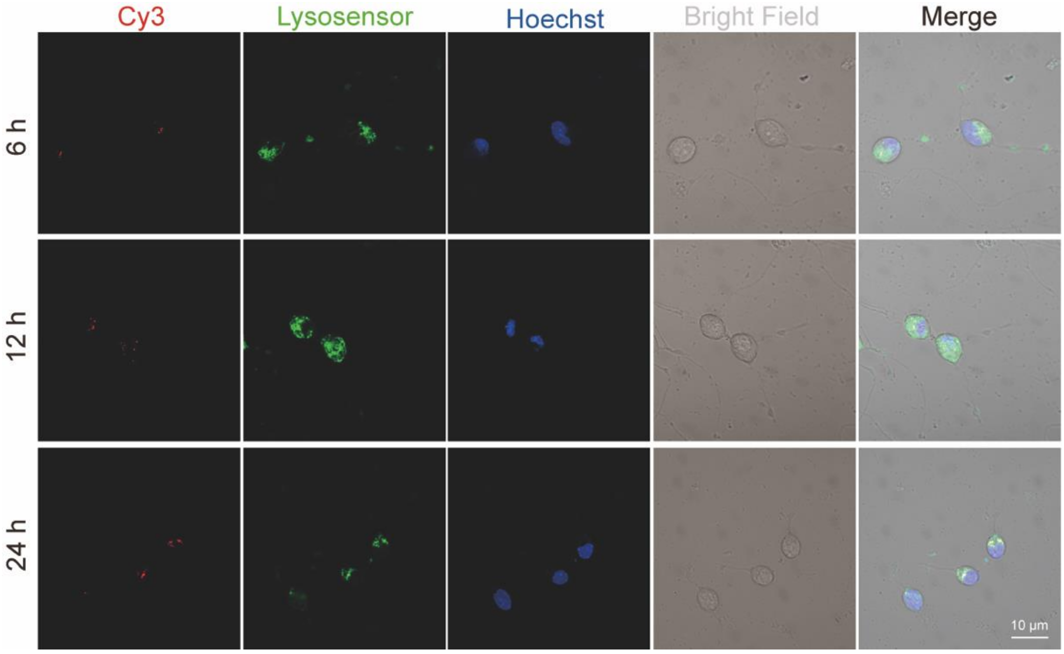

Figure S4. Uptake of miR-MSN by primary neurons in vitro. Representative fluorescence images of miR-MSN, lysosomes, nuclei, bright-field and merged images of neurons cultured with miR-MSN for 6, 12, and 24 h are shown. The miRNA was stained with Cy3 (red), lysosomes with Lysosensor (green), and nuclei with Hoechst (blue). Scale bar: 10 μm.

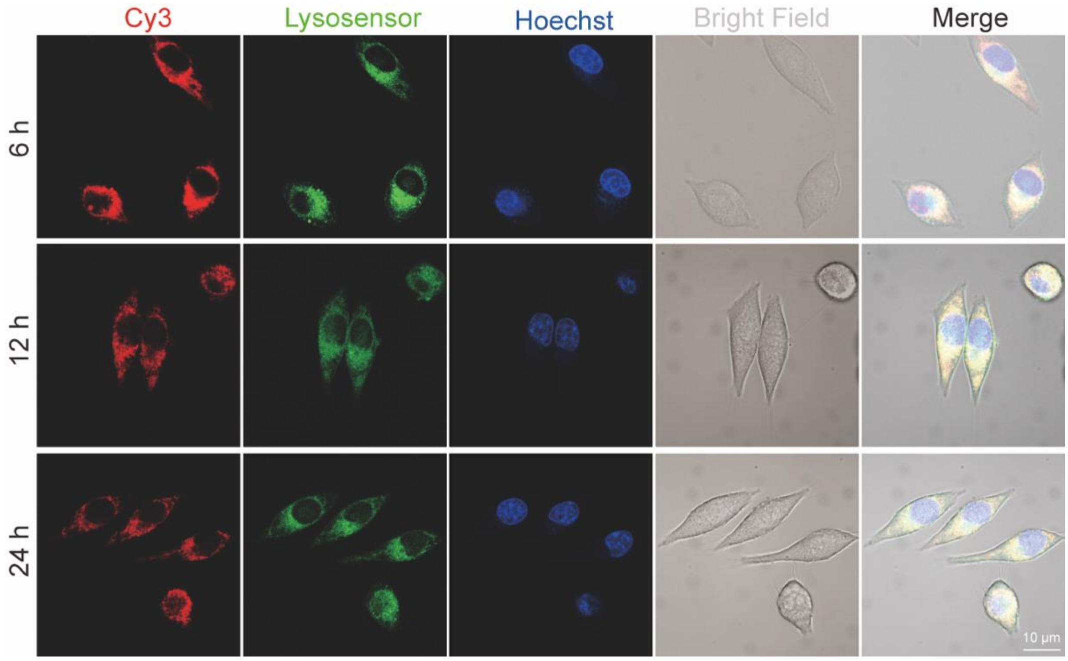

Figure S5. Phagocytosis of miR-MSN by BV2 in vitro. Representative fluorescence images of miR-MSN, lysosomes, and nuclei; bright-field images and merged images of BV2 cultured with miR-MSN for 6, 12, and 24 h are shown. The miRNA was stained with Cy3 (red), lysosomes with Lysosensor (green), and nuclei with Hoechst (blue). Scale bar: 10 μm.

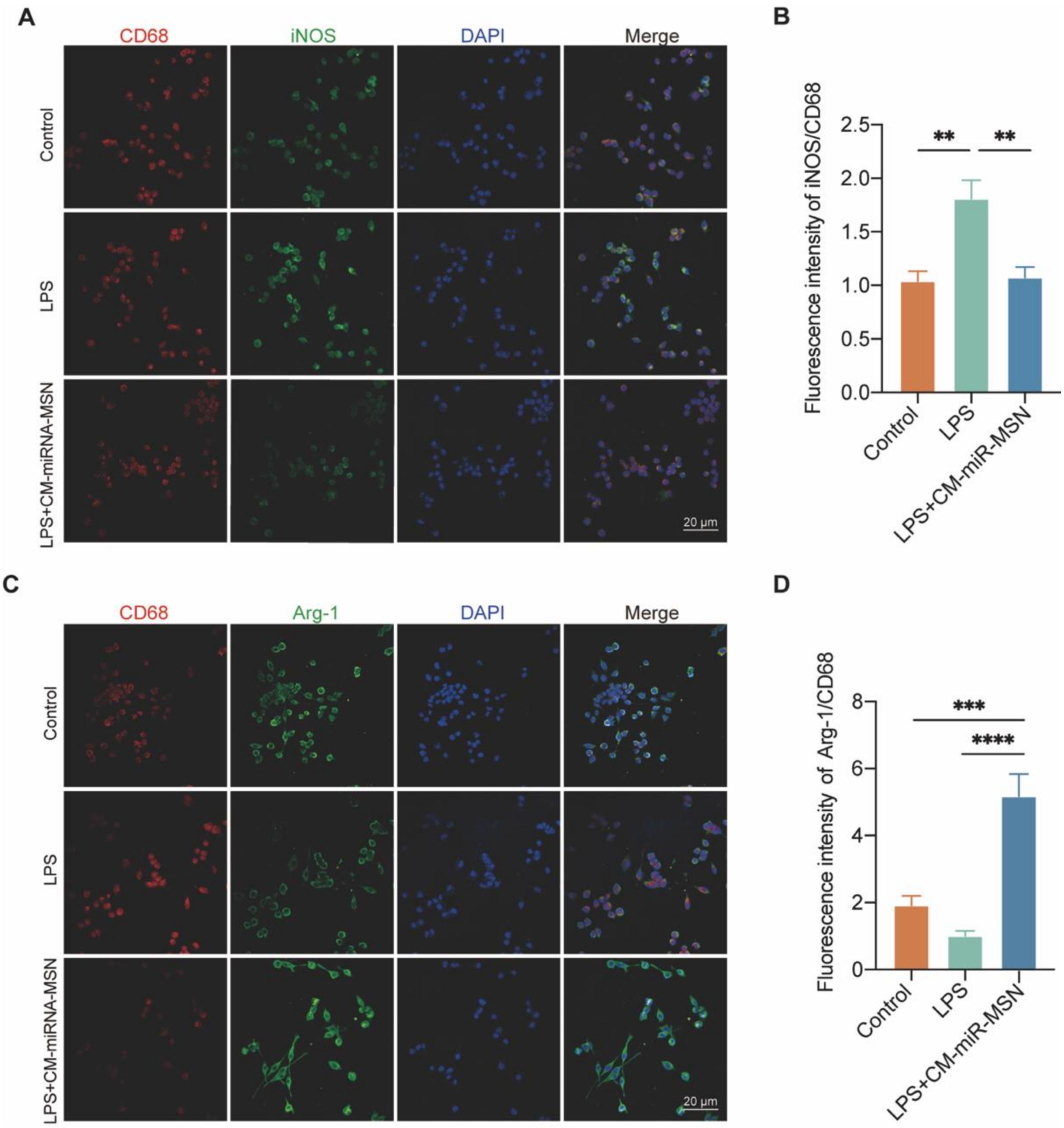

Figure S6. Immunofluorescence staining suggests CM-miR-MSN downregulates iNOS and upregulates Arg-1 in lipopolysaccharide (LPS)-stimulated BV2. A) Immunofluorescence staining of CD68 and iNOS in control, LPS, and LPS + CM-miR-MSN group. Scale bar: 20 μm. B) Quantification of iNOS/CD68 fluorescence intensity in (A). We performed statistical analysis using one-way analysis of variance [ANOVA]; n = 3, **p < 0.01. C) Immunofluorescence staining of CD68 and Arg-1 in each group. Scale bar: 20 μm. D) Quantification of Arg-1/CD68 fluorescence intensity in (C). We performed statistical analysis using one-way ANOVA; n = 3, ***p < 0.001, and ****p < 0.0001.

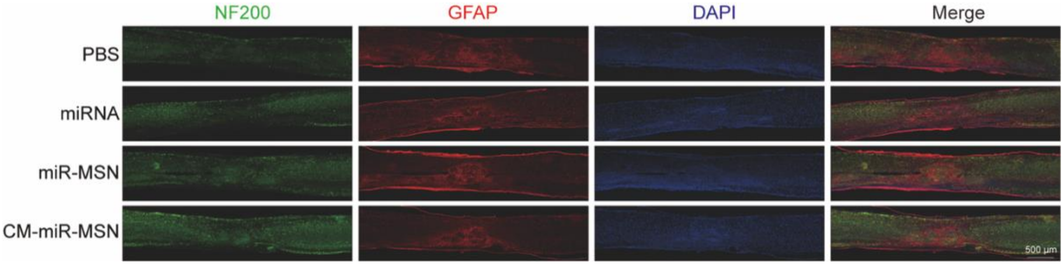

Figure S7. Immunofluorescence staining of NF200-positive neuro-filaments and GFAP-positive astrocytes in sagittal sections of the injured area (n = 6). Scale bar: 500 μm.

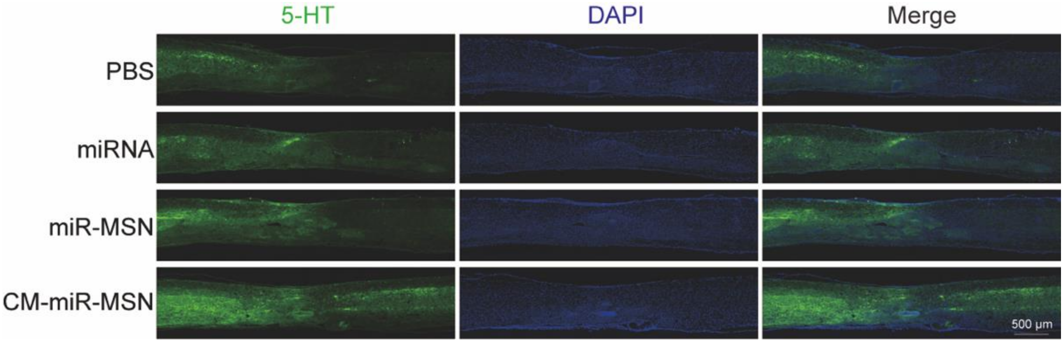

Figure S8. Representative images of regenerating serotonergic axons beyond the lesion site (n = 6). Scale bar: 500 μm.

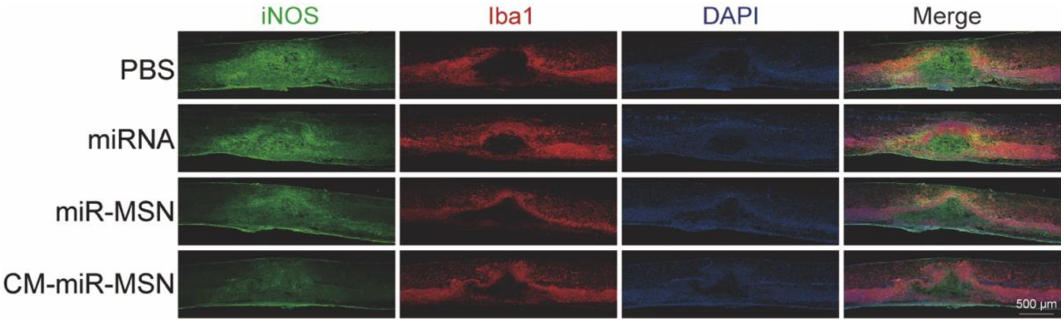

Figure S9. Immunofluorescence staining of iNOS and Iba1 in each group at day 7 after spinal cord injury (n = 3). Scale bar: 500 μm.

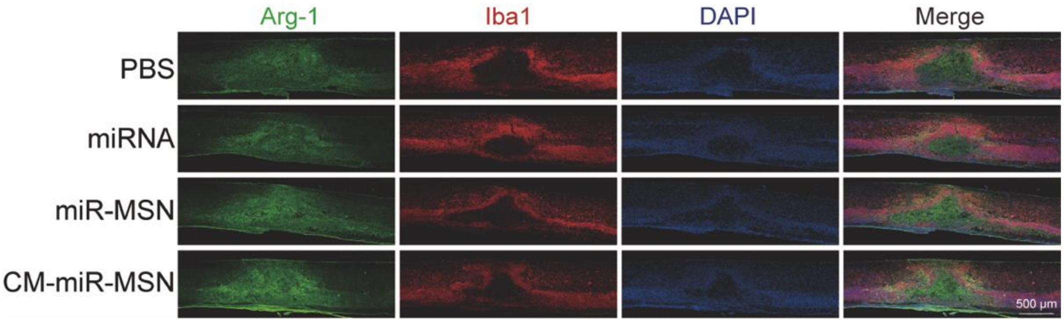

Figure S10. Immunofluorescence staining of Arg-1 and Iba1 in each group at day 7 after spinal cord injury (n = 3). Scale bar: 500 μm.

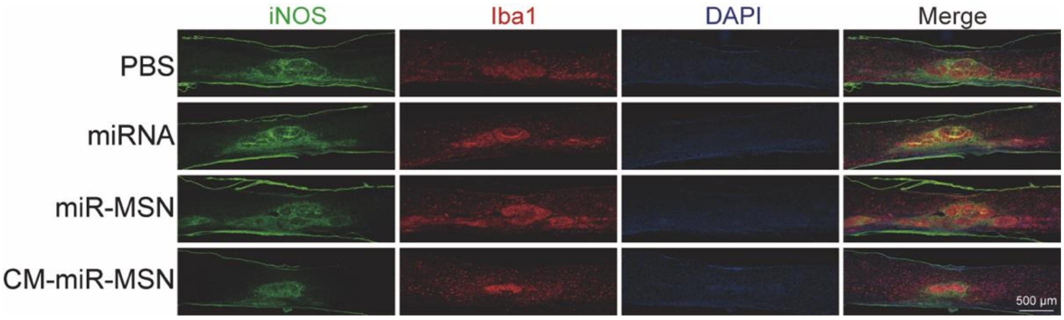

Figure S11. Immunofluorescence staining of iNOS and Iba1 in each group at day 28 after spinal cord injury (n = 6). Scale bar: 500 μm.

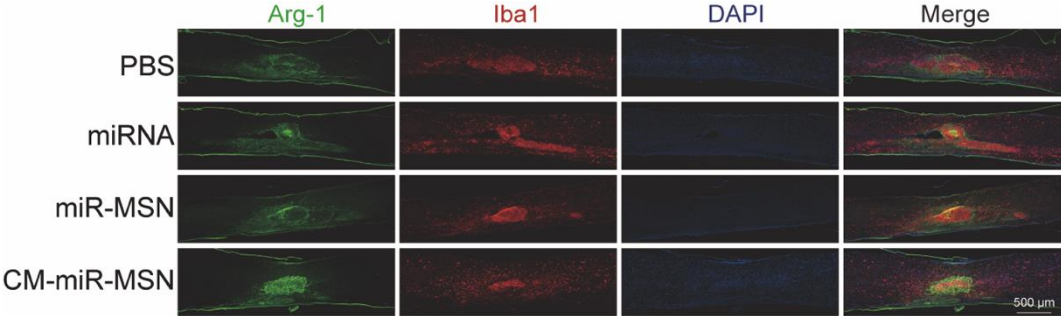

Figure S12. Immunofluorescence staining of Arg-1 and Iba1 in each group at day 28 after spinal cord injury (n = 6). Scale bar: 500 μm.

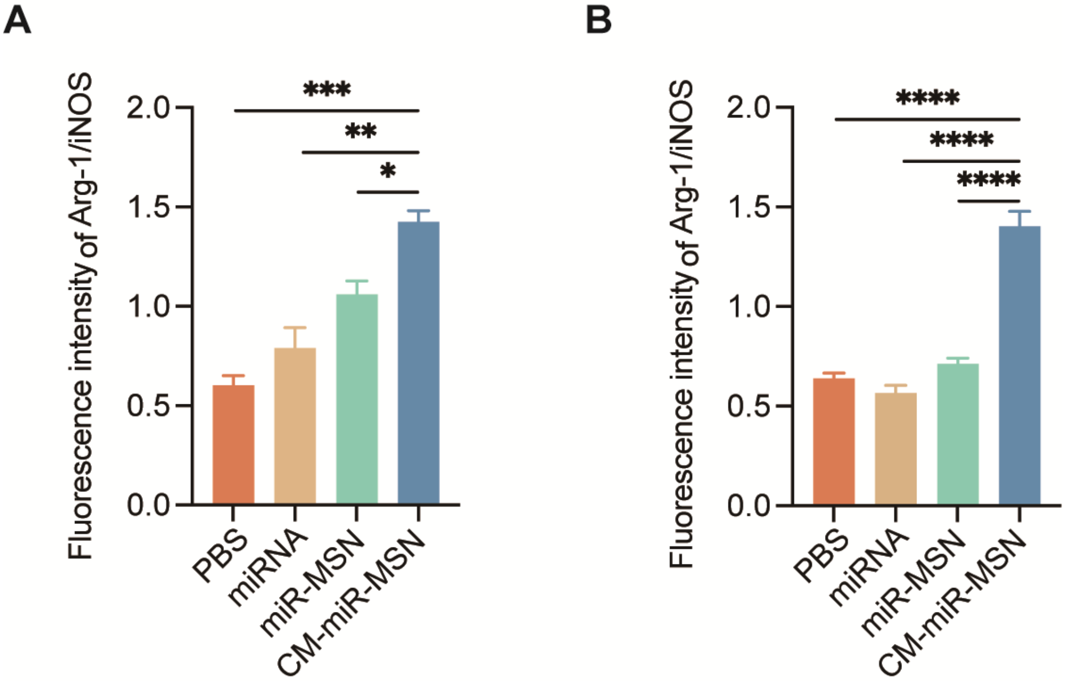

Figure S13. A) Quantification of Arg-1/iNOS fluorescence on day 7 post-injury performed using one-way ANOVA (n = 3); *p < 0.05, **p < 0.01, and ***p < 0.001. B) Quantification of Arg-1/iNOS fluorescence intensity on day 28 post-injury (n = 6). Statistical analysis was performed using one-way ANOVA; ****p < 0.0001.

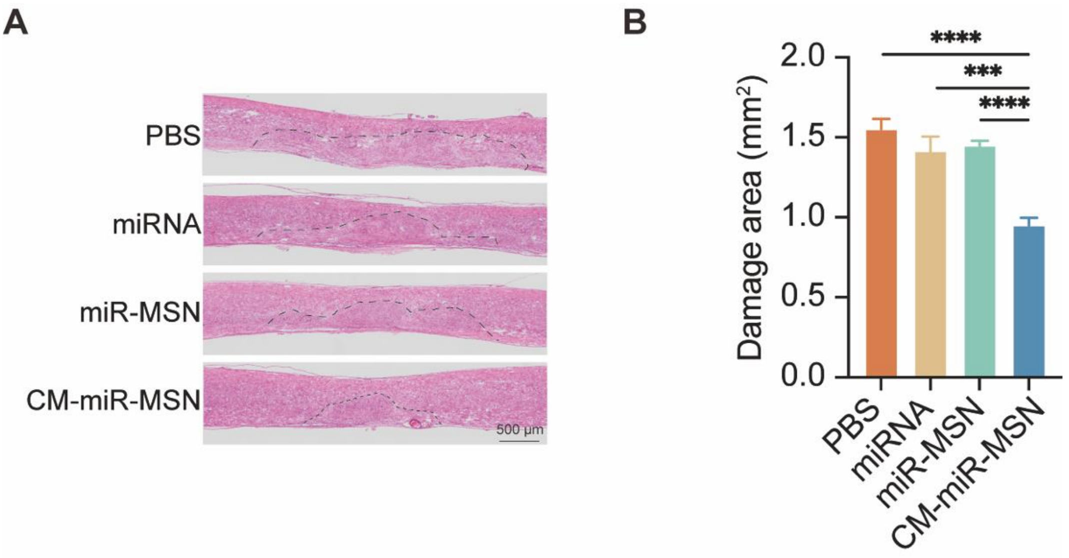

Figure S14. Hematoxylin and eosin (H&E) staining of sagittal spinal cord sections at day 28 after SCI (n = 6). Scale bars: 500 µm. A) Representative H&E staining sections. B) Quantification of damaged area in each group. Statistical analysis was performed using one-way analysis of variance; ***p < 0.001 and ****p < 0.0001.

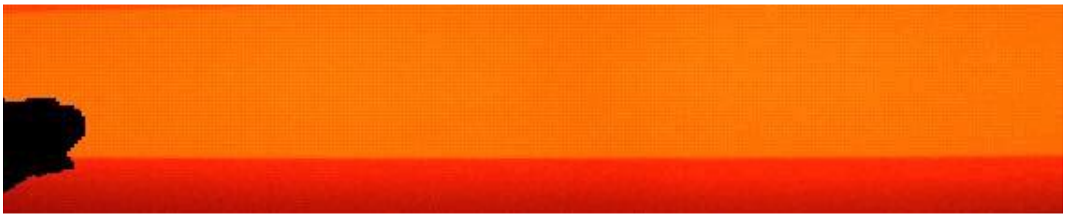

VideoS1

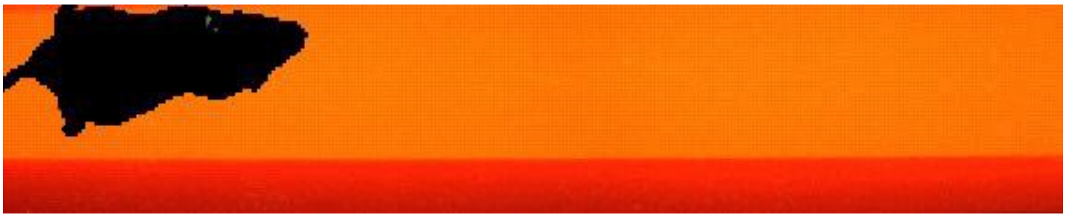

VideoS2

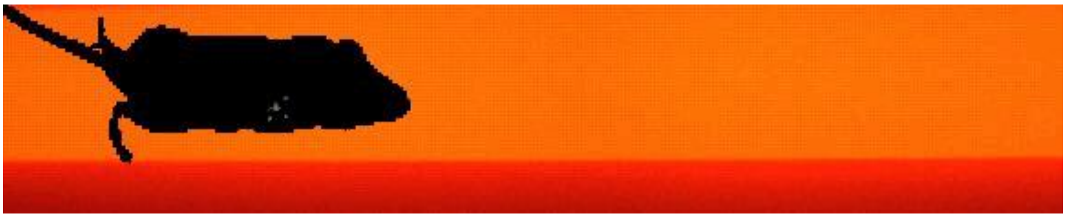

VideoS3

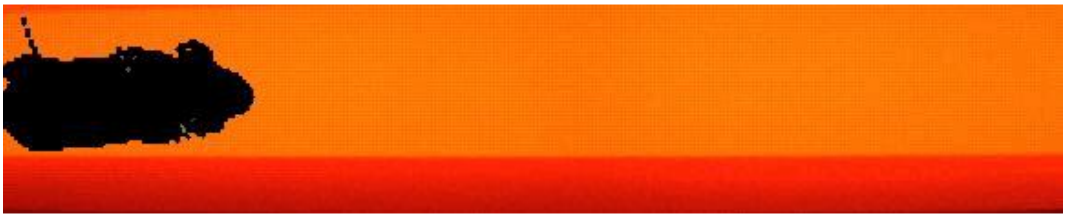

VideoS4

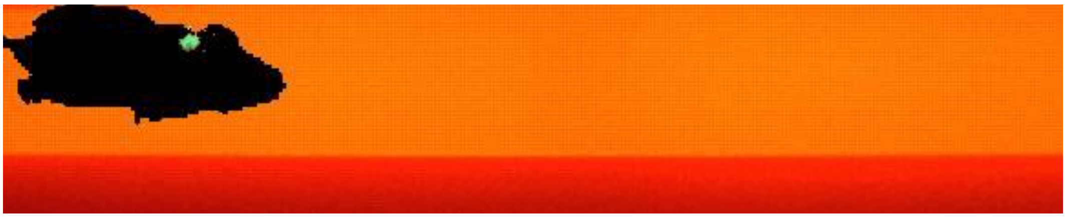

VideoS5

VideoS1-5. Videos of mouse movements while the mice passed through the illuminated ceiling.

VideoS1) Movement of a normal mouse.

VideoS2) Movement of an SCI mouse treated with PBS.

VideoS3) Movement of an SCI mouse treated with miRNA.

VideoS4) Movement of an SCI mouse treated with miR-MSN.

VideoS5) Movement of an SCI mouse treated with CM-miR-MSN.
